# Long-term variations of urban–Rural disparities in infectious disease burden of over 8.44 million children, adolescents, and youth in China from 2013 to 2021: An observational study

**DOI:** 10.1371/journal.pmed.1004374

**Published:** 2024-04-12

**Authors:** Li Chen, Yi Xing, Yi Zhang, Junqing Xie, Binbin Su, Jianuo Jiang, Mengjie Geng, Xiang Ren, Tongjun Guo, Wen Yuan, Qi Ma, Manman Chen, Mengjie Cui, Jieyu Liu, Yi Song, Liping Wang, Yanhui Dong, Jun Ma

**Affiliations:** 1 Institute of Child and Adolescent Health, School of Public Health, Peking University; National Health Commission Key Laboratory of Reproductive Health, Beijing, China; 2 Centre for Statistics in Medicine, NDORMS, University of Oxford, Oxford, United Kingdom; 3 School of Population Medicine and Public Health, Chinese Academy of Medical Sciences/ Peking Union Medical College, Beijing, China; 4 Division of Infectious Disease Control and Prevention, Key Laboratory of Surveillance and Early Warning on Infectious Disease, Chinese Center for Disease Control and Prevention, Beijing, China; Washington University School of Medicine, UNITED STATES

## Abstract

**Background:**

An accelerated epidemiological transition, spurred by economic development and urbanization, has led to a rapid transformation of the disease spectrum. However, this transition has resulted in a divergent change in the burden of infectious diseases between urban and rural areas. The objective of our study was to evaluate the long-term urban–rural disparities in infectious diseases among children, adolescents, and youths in China, while also examining the specific diseases driving these disparities.

**Methods and findings:**

This observational study examined data on 43 notifiable infectious diseases from 8,442,956 cases from individuals aged 4 to 24 years, with 4,487,043 cases in urban areas and 3,955,913 in rural areas. The data from 2013 to 2021 were obtained from China’s Notifiable Infectious Disease Surveillance System. The 43 infectious diseases were categorized into 7 categories: vaccine-preventable, bacterial, gastrointestinal and enterovirus, sexually transmitted and bloodborne, vectorborne, zoonotic, and quarantinable diseases. The calculation of infectious disease incidence was stratified by urban and rural areas. We used the index of incidence rate ratio (IRR), calculated by dividing the urban incidence rate by the rural incidence rate for each disease category, to assess the urban–rural disparity.

During the nine-year study period, most notifiable infectious diseases in both urban and rural areas exhibited either a decreased or stable pattern. However, a significant and progressively widening urban–rural disparity in notifiable infectious diseases was observed. Children, adolescents, and youths in urban areas experienced a higher average yearly incidence compared to their rural counterparts, with rates of 439 per 100,000 compared to 211 per 100,000, respectively (IRR: 2.078, 95% CI [2.075, 2.081]; *p* < 0.001).

From 2013 to 2021, this disparity was primarily driven by higher incidences of pertussis (IRR: 1.782, 95% CI [1.705, 1.862]; *p* < 0.001) and seasonal influenza (IRR: 3.213, 95% CI [3.205, 3.220]; *p* < 0.001) among vaccine-preventable diseases, tuberculosis (IRR: 1.011, 95% CI [1.006, 1.015]; *p* < 0.001), and scarlet fever (IRR: 2.942, 95% CI [2.918, 2.966]; *p* < 0.001) among bacterial diseases, infectious diarrhea (IRR: 1.932, 95% CI [1.924, 1.939]; *p* < 0.001), and hand, foot, and mouth disease (IRR: 2.501, 95% CI [2.491, 2.510]; *p* < 0.001) among gastrointestinal and enterovirus diseases, dengue (IRR: 11.952, 95% CI [11.313, 12.628]; *p* < 0.001) among vectorborne diseases, and 4 sexually transmitted and bloodborne diseases (syphilis: IRR 1.743, 95% CI [1.731, 1.755], *p* < 0.001; gonorrhea: IRR 2.658, 95% CI [2.635, 2.682], *p* < 0.001; HIV/AIDS: IRR 2.269, 95% CI [2.239, 2.299], *p* < 0.001; hepatitis C: IRR 1.540, 95% CI [1.506, 1.575], *p* < 0.001), but was partially offset by lower incidences of most zoonotic and quarantinable diseases in urban areas (for example, brucellosis among zoonotic: IRR 0.516, 95% CI [0.498, 0.534], *p* < 0.001; hemorrhagic fever among quarantinable: IRR 0.930, 95% CI [0.881, 0.981], *p* = 0.008). Additionally, the overall urban–rural disparity was particularly pronounced in the middle (IRR: 1.704, 95% CI [1.699, 1.708]; *p* < 0.001) and northeastern regions (IRR: 1.713, 95% CI [1.700, 1.726]; *p* < 0.001) of China. A primary limitation of our study is that the incidence was calculated based on annual average population data without accounting for population mobility.

**Conclusions:**

A significant urban–rural disparity in notifiable infectious diseases among children, adolescents, and youths was evident from our study. The burden in urban areas exceeded that in rural areas by more than 2-fold, and this gap appears to be widening, particularly influenced by tuberculosis, scarlet fever, infectious diarrhea, and typhus. These findings underscore the urgent need for interventions to mitigate infectious diseases and address the growing urban–rural disparity.

## Abstract 2

Li Chen, Yi Xing, and colleagues examine the long-term urban-rural disparities in 43 infectious diseases among children, adolescents, and youths in China.

## Author summary

**Why was this study done?:**

➢ Despite national health priorities and immunization programs, the urban–rural disparity in infectious diseases in children and adolescents is often overlooked.
➢ To our knowledge, while some studies have evaluated infectious diseases burdens in children, adolescents, and youths, none have conducted a comprehensive comparison of disease patterns between urban and rural areas in China or other countries.

**What did the researchers do and find?:**

- ➢ We used the incident cases of infectious diseases aged 4 to 24 years old from China to study the urban–rural disparity in infectious diseases and its long-term change over time.
- ➢ The burden of infectious diseases in urban areas was more than double that of rural areas, and this disparity widened over time, particularly for tuberculosis and scarlet fever among vaccine-preventable diseases, and infectious diarrhea among gastrointestinal and enterovirus diseases.
- ➢ Among Chinese youth, overall infectious diseases were unequally distributed, with vaccine-preventable diseases showing the highest inequality.

**What do these findings mean?:**

- ➢ The substantial burden of infectious diseases in urban areas, underscores the unique health challenges faced by urban populations.
- ➢ The urban–rural disparities and inequality underscore the need for nuanced strategies in addressing infectious diseases, influenced by factors like socioeconomic development and urbanization, across diverse disease types and geographical contexts.
- ➢ Study limitations involve using annual average population data without considering population mobility for incidence calculations.

## Introduction

Infectious diseases persist as an enduring global challenge, marked by their swift dissemination on a worldwide scale, further accelerated by the heightened mobility of populations [1–4]. The primary drivers of global population mobility have shifted from war and colonization to trade resulting in accelerated globalization and urbanization worldwide [5], including a particularly notable rise in the number of large and mega cities [6–9]. Urban areas, surpassing rural ones in population since 2009, are projected to house 60% of the global population by 2030 [10,11]. In China, rapid urbanization since the 1990s has led to a significant rural-to-urban migration, with more than half of its population residing in urban areas as of 2011 [12,13]. This concentration in urban centers, along with international trade, elevates the risk of infectious disease outbreaks globally, underscoring the importance of examining urban–rural disparities in disease burden. Despite the critical need for such analysis, a comprehensive systematic review of this disparity is, to our knowledge, currently lacking. While 2 previous studies in the United Kingdom (2016 to 2017) and China (1990 to 2010) reported on the incidence of infectious diseases in urban and rural areas, they presented divergent findings [14,15]. The UK study identified a higher incidence of 6 infectious disease categories in urban areas [15], while the Chinese investigation observed a shift from higher overall incidence in rural areas before 2005 to higher incidence in urban areas after 2005. This alignment in findings highlights a general trend of increasing infectious disease incidence in urban settings, though the timing and magnitude of these shifts may vary across different countries and time periods. To our knowledge, no study to date has systematically addressed disparities in the complete spectrum of infectious diseases between urban and rural areas, especially concerning vulnerable demographic groups such as children and adolescents.

Studying urban–rural disparities in infectious diseases is pivotal for comprehending and remedying the unequal burden of disease across various populations [16]. Rapid urbanization, marked by a significant migration of individuals to urban centers, results in increased population density, closer living quarters, and heightened interpersonal interactions [5,17]. These factors create an environment conducive to the rapid spread of infectious diseases. Thus, urbanization plays an important role in exacerbating or mitigating health inequalities between urban and rural areas. The association between urbanization and health is intricate, with several studies highlighting its impact on life expectancy, especially in high versus low-income countries [18]. Urbanization has led to mixed outcomes in health, particularly for children and adolescents [19]. While there has been a notable decline in infectious disease mortality in these younger populations, contributing to increased life expectancy [20,21], this is not evident in all communities. In impoverished and marginalized communities, life expectancy has stagnated or even declined [22–24]. Despite the overall decrease in incidence and mortality from infectious diseases among children and adolescents in recent years [25,26], these diseases remain a significant public health concern as they evolve rapidly with urbanization. Future public health strategies must focus on developing targeted measures to control infectious diseases in children and adolescents based on population evolution in urban and rural settings. The uncertainty surrounding the long-term evolution of the urban–rural gap in infectious diseases complicates the formulation of targeted measures to address diverse population needs. Narrowing the disparity in infectious disease burdens between urban and rural areas is crucial, alongside efforts to mitigate health inequality.

There may be some basis for differences in the role of urbanization in causing different types of infectious diseases in urban and rural settings [5,17]. Rural regions often contend with suboptimal living conditions, exacerbated by livestock breeding and frequent human–wildlife–livestock interactions, leading to increased susceptibility to vector-borne and zoonotic infectious diseases [2]. While waterborne and vector-borne diseases, historically linked to poor sanitation and agricultural practices, are on the decline in urban settings due to improved access to clean water and sanitation facilities, infectious diseases associated with close interpersonal contact and urban lifestyles are gaining prominence [26]. In addition, the interplay between infectious diseases and urbanization is multifaceted. Rural areas may encounter challenges with vector-borne diseases due to poverty-related factors, while urban environments, characterized by high population density and lifestyle-related issues, may experience an uptick in respiratory infections. Furthermore, urban locales enjoy the advantages of advanced healthcare infrastructure, which culminate in more effective disease surveillance and reporting mechanisms [26]. This indicates the distinct challenges faced by urban and rural areas in addressing infectious diseases. These disparities hold significant public health implications, shaping disease prevention and control strategies, resource allocation, and healthcare policy-making [27]. However, amidst the backdrop of rapid urbanization, there remains a paucity of evidence regarding the enduring urban–rural disparities stemming from infectious diseases and the specific disease types that propel the evolution of these discrepancies.

As global urbanization progresses, understanding disparities in infectious diseases among children and adolescents in urban and rural areas becomes increasingly vital. To address this gap, we conducted a nine-year (2013 to 2021) observational analysis of notified infectious diseases among 4- to 24-year-olds in China, examining incidence disparities between rural and urban areas and exploring various specific diseases influencing urban–rural disparities in infectious disease incidence.

## Methods

### Data collection

Data in the study was drawn from the China Information System for Disease Control and Prevention (CISDCP), which is established in 2003 and is an internet-based, long-term surveillance system that covers more than 85% of all health facilities in China, and its details were reported in the previous studies [26,28,29]. The CISDCP includes the identification of all notifiable infectious diseases and the number of cases for each. The system requires that cholera, plague, anthrax, and severe acute respiratory syndrome (SARS) be reported within 2 h, and all other infectious diseases within 24 h. The vertical reporting structure of CISDCP was presented in **S1 Fig**. When a probable, clinical, or laboratory-confirmed case is diagnosed, doctors must complete a standard card and submit it to CISDCP within 24 h. Local epidemiologists then conduct a field investigation using a standard form that includes basic demographic data, case classification, dates of symptom onset, diagnosis, and clinical outcome including death (if applicable).

For this study, we included children and adolescents aged 4 to 24 years old with confirmed records of 43 notifiable infectious diseases from 2013 to 2021, excluding cases from Hong Kong, Macao, Taiwan of China, and other countries with unverified records. The selected age range was determined by considering the typical entry age of 4 years old for kindergarten, aligning with the educational phase’s monitoring scope. Furthermore, the upper limit of 24 years old corresponds to the age of university students and is in line with the United Nations definition of “youth” (15 to 24) [30]. This age range was chosen to ensure comprehensive coverage of relevant age groups for the study, making our research findings more pertinent to the specific circumstances in China and facilitating broader applicability to similar countries. The Chinese Centers for Disease Control and Prevention (CDC) is required to meticulously scrutinize pertinent details within verified disease cases, including but not limited to occupation, age, geographical location, and date of diagnosis. The classification of infectious disease cases as verified necessitates a comprehensive examination to ascertain the absence of omissions and logical inconsistencies in the dataset. The study did not necessitate ethical approval as it solely utilized data that was both de-identified and publicly accessible, thereby exempting it from Institutional Review Board (IRB) or ethics committee review processes. In this observational study, we included infectious disease cases notified to CISDCP from 2013 to 2021. For all 43 infectious diseases (refer to **Table 1**), we further categorized them into 7 groups, including vaccine-preventable, bacterial, gastrointestinal and enterovirus, sexually transmitted and bloodborne, vectorborne, zoonotic, and quarantinable diseases.

**Table 1:** The overall disparity in incidence of total 43 notifiable infectious diseases by category and specific diseases among children, adolescents, and youth aged 4 to 24 years in urban and rural areas.

Category	Urban		Rural		IRR (urban vs. rural)		
	Cases (*n*)*	Yearly incidence^#^	Cases (*n*)*	Yearly incidence^#^	95% CI		*p* value
Overall	4,487,043	439.360	3,955,913	211.464	2.078	2.075, 2.081	<0.001
Vaccine preventable	2,506,822	246.526	2,002,540	107.360	2.297	2.293, 2.301	<0.001
SI	1,750,418	173.499	993,206	54.017	3.213	3.205, 3.220	<0.001
Mumps	565,432	54.583	655,213	34.619	1.577	1.571, 1.582	<0.001
Hepatitis B	153,707	14.824	301,065	15.906	0.932	0.926, 0.938	<0.001
Rubella	22,066	2.148	30,019	1.601	1.341	1.318, 1.365	<0.001
Hepatitis A	5,106	0.490	11,140	0.584	0.839	0.811, 0.867	<0.001
Measles	6,157	0.589	7,839	0.411	1.434	1.387, 1.482	<0.001
Pertussis	3,916	0.390	4,016	0.219	1.782	1.705, 1.862	<0.001
Hepatitis D	20	0.003	42	0.003	1.032	0.606, 1.758	0.908
Diphtheria	-	-	-	-	-	-	-
NT	-	-	-	-	-	-	-
Poliomyelitis	-	-	-	-	-	-	-
**Bacteria**	**441,820**	**42.760**	**616,794**	**32.731**	**1.306**	**1.301, 1.311**	**<0.001**
TB	288,974	27.980	521,582	27.684	1.011	1.006, 1.015	<0.001
SF	152,617	14.757	94,624	5.016	2.942	2.918, 2.966	<0.001
MM	141	0.014	345	0.018	0.746	0.613, 0.907	0.003
Leprosy	88	0.009	243	0.013	0.661	0.518, 0.844	0.001
**Gastrointestil and enterovirus**	**1,175,199**	**114.586**	**1,002,018**	**53.462**	**2.143**	**2.138, 2.149**	**<0.001**
HFMD	604,020	58.687	441,399	23.468	2.501	2.491, 2.510	<0.001
ID	476,759	46.787	451,450	24.223	1.932	1.924, 1.939	<0.001
Dysentery	65,398	6.307	62,843	3.316	1.902	1.881, 1.923	<0.001
AHC	21,242	2.054	35,709	1.894	1.085	1.067, 1.104	<0.001
T/P	7,780	0.751	10,617	0.561	1.339	1.300, 1.379	<0.001
**Sexually transmitted and bloodborne**	**341,445**	**33.360**	**303,509**	**16.247**	**2.053**	**2.043, 2.063**	**<0.001**
Syphilis	156,149	15.274	163,520	8.762	1.743	1.731, 1.755	<0.001
Gonorrhea	121,025	11.853	82,982	4.458	2.658	2.635, 2.682	<0.001
HIV/AIDS	50,291	4.891	40,480	2.156	2.269	2.239, 2.299	<0.001
Hepatitis C	13,980	1.342	16,527	0.871	1.540	1.506, 1.575	<0.001
**Vectorborne**	**12,565**	**1.234**	**6,418**	**0.352**	**3.501**	**3.397, 3.608**	**<0.001**
Dengue	10,770	1.039	1,441	0.087	11.952	11.313, 12.628	<0.001
JE	631	0.06	2,183	0.115	0.527	0.482, 0.576	<0.001
Typhus	536	0.052	1,554	0.082	0.633	0.574, 0.698	<0.001
Malaria	289	0.028	598	0.031	0.886	0.770, 1.020	0.092
SM	289	0.050	533	0.031	1.584	1.372, 1.827	<0.001
Kala, azar	50	0.005	109	0.006	0.833	0.596, 1.164	0.285
Filariasis	-	-	-	-	-	-	-
**Zoonotic**	**7,213**	**0.701**	**20,739**	**1.104**	**0.636**	**0.620, 0.654**	**<0.001**
Brucellosis	4,003	0.390	14,199	0.757	0.516	0.498, 0.534	<0.001
Hepatitis E	2,305	0.223	2,509	0.133	1.680	1.587, 1.777	<0.001
HD	715	0.069	3,296	0.175	0.396	0.365, 0.430	<0.001
Rabies	129	0.012	422	0.022	0.559	0.459, 0.680	<0.001
Anthrax	16	0.002	178	0.009	0.184	0.110, 0.306	<0.001
Leptospirosis	34	0.003	128	0.007	0.487	0.333, 0.710	<0.001
H7N9	11	0.002	7	0.001	2.148	0.833, 5.541	0.114
H5N1	-	-	-	-	-	-	-
SARS	-	-	-	-	-	-	-
**Quarantinable**	**1,979**	**0.193**	**3,895**	**0.208**	**0.932**	**0.883, 0.984**	**0.011**
HF	1,970	0.192	3,890	0.207	0.930	0.881, 0.981	0.008
Cholera	9	0.001	5	0.001	1.899	0.636, 5.665	0.250
Plague	-	-	-	-	-	-	-

*Total numbers from 2013 to 2021.

^#^Average incidence 2013 to 2021; -, no cases.

SI, seasonal influenza; NT, neonatal tetanus; TB, tuberculosis; SF, scarlet fever; MM, meningococcal meningitis; T/P, typhoid fever and paratyphoid fever; HFMD, hand, foot, and mouth disease; AHC, acute hemorrhagic conjunctivitis; ID, infectious diarrhea; AIDS, acquired immune deficiency syndrome; SM, schistosomiasis; JE, Japanese encephalitis; HD, hydatid disease; SARS, severe acute respiratory syndrome; HF, hemorrhagic fever; IRR, incidence rate ratio.

### Urban and rural definition

According to the administrative divisions and economic characteristics of China, there are a total of 34 provinces, 333 prefecture-level cities, and 2,843 districts/counties [31]. Urban and rural classification is based on the 2,843 districts/counties, of which 973 districts are classified as urban areas and the rest as rural areas. In this study, we defined the main economic and densely populated areas in each prefecture-level city as urban areas (973 districts) [31], which typically have higher economic and educational levels and higher population density. The remaining counties in each prefecture-level city (1,870 counties) [31], excluding the economic and densely populated areas, are defined as rural areas, which typically cover a larger land area but have lower population density and lower economic levels. The distribution of urban and rural areas was shown in **S2 Fig**, along with the schematic diagram of the 3 levels of administrative division in China, based on Sichuan province and Chengdu city.

### Geographic classification

Considering the substantial economic disparities among the provinces, we divided the 31 provinces of China into 4 regions: eastern, central, western, and northeastern, based on their latitude and longitude, as well as administrative region division. The eastern region is located in the coastal area with a higher economic level, followed by the central region, while the western region has a relatively lagging economy [32]. We obtained the population data for each district/county from the 2020 Chinese Population Census Yearbook. By using administrative divisions, we were able to gather data on the population of males and females, as well as different age groups, for each prefecture-level city, province, and the entire country, which we considered to be the total population for each respective area.

### Statistical analysis

Aligned with the research objectives, this investigation hypothesizes that there is disparity in infectious disease incidence between urban and rural areas. Additionally, it posits notable variations in the distribution of diverse categories and individual infectious diseases across these areas, with heightened heterogeneity within urban areas. At the outset of the research, a comprehensive analysis strategy was prospectively designed and produced for the current investigation (**S1 Text**).

To examine the urban–rural disparities in disease incidence, we calculated the incidence rate by dividing the number of reported cases by the total population size for both overall infectious diseases and specific disease categories in both urban and rural areas. For most infectious diseases, we used integer values in the main text. Infectious diseases with lower incidence, like vectorborne, zoonotic, and quarantinable diseases, retained 1 decimal place. We then determined the urban–rural incidence rate ratio (IRR) by dividing the urban incidence rate by the rural incidence rate for each disease category. The incidence rates in urban and rural areas and the IRR were used to quantify urban–rural disparities. We present unadjusted analyses in this study, mainly because the dataset closely represents the entire Chinese population of children and adolescents aged 4 to 24, akin to census data, and lacked additional variables for adjustment. Nevertheless, to ensure a thorough presentation of the findings, subgroup analyses were undertaken, delineating variations across different regions and between sexes. We assessed the urban–rural disparities between 2013 and 2021 for each quartile of socioeconomic indicators, each region group, and each province. We also evaluated the relationship between changes in socioeconomic indicators and differences in overall incidence and disease classification between urban and rural areas (**S1 Methods and S2 Methods**). To identify the long-term patterns in the disease incidence and IRR, we utilized Joinpoint regression models, which estimated annual percentage changes and tested for significance using a Z test. We classified the patterns as either increasing or decreasing, defined by the slope of the annual percent change (APC) and its statistical significance (*p* < 0.05). Patterns where the APC was not statistically significant (*p* ≥ 0.05) were characterized as stable, indicating that the incidence or IRR either remained constant or varied without discernible statistical significance.

All statistical analyses were performed using the R program (version 4.1.1) [33], and the R packages “gam” and “ineq” were applied to perform the GAMs and Lorenz curve. Joinpoint regression was conducted using Joinpoint software (version 4.3.1) [34] to evaluate long-term patterns in incidence and IRR.

## Results

### Overall urban–rural disparity of notifiable infectious diseases

From 2013 to 2021, a total of 8,442,956 cases of 43 notifiable infectious diseases were reported in Chinese children, adolescents, and youth, with 4,487,043 cases from urban areas and an average yearly incidence of 439 per 100,000, and 3,955,913 cases from rural areas and an average yearly incidence of 211 per 100,000 (**Table 1, S3** and **S4 Figs**). The age range and sex distribution for each infectious disease is presented in **S1 Table**. Overall, a higher burden of notifiable infectious diseases was observed in children, adolescents, and youths in urban areas, with an IRR of 2.078 (95% CI: 2.075, 2.081; *p* < 0.001) (**Table 1**). Among the 7 classifications of infectious diseases, zoonotic diseases (IRR of 0.636 (95% CI [0.620, 0.654]; *p* < 0.001; 0.7 per 100,000 in urban areas versus 1.1 per 100,000 in rural areas) and quarantinable diseases (IRR 0.932; 95% CI [0.883, 0.984]; *p* = 0.011; 0.2 versus 0.2 per 100,000) were higher in rural areas than in urban areas. Infectious diseases of all other classifications were higher in urban areas, including vaccine-preventable diseases (IRR = 2.297; 95% CI [2.293, 2.301]; *p* < 0.001; 247 versus 107 per 100,000), bacterial diseases (IRR = 1.306; 95% CI [1.301, 1.311]; *p* < 0.001; 43 versus 33 per 100,000), gastrointestinal and enterovirus diseases (IRR = 2.143; 95% CI [2.138, 2.149]; *p* < 0.001; 115 versus 53 per 100,000), sexually transmitted and bloodborne diseases (IRR = 2.053; 95% CI [2.043, 2.063]; *p* < 0.001; 33 versus 16 per 100,000), and vectorborne diseases (IRR = 3.501; 95% CI [3.397, 3.608]; *p* < 0.001; 1.2 versus 0.4 per 100,000) (**Table 1**).

### Overall urban–rural disparity by year

**Fig 1:**
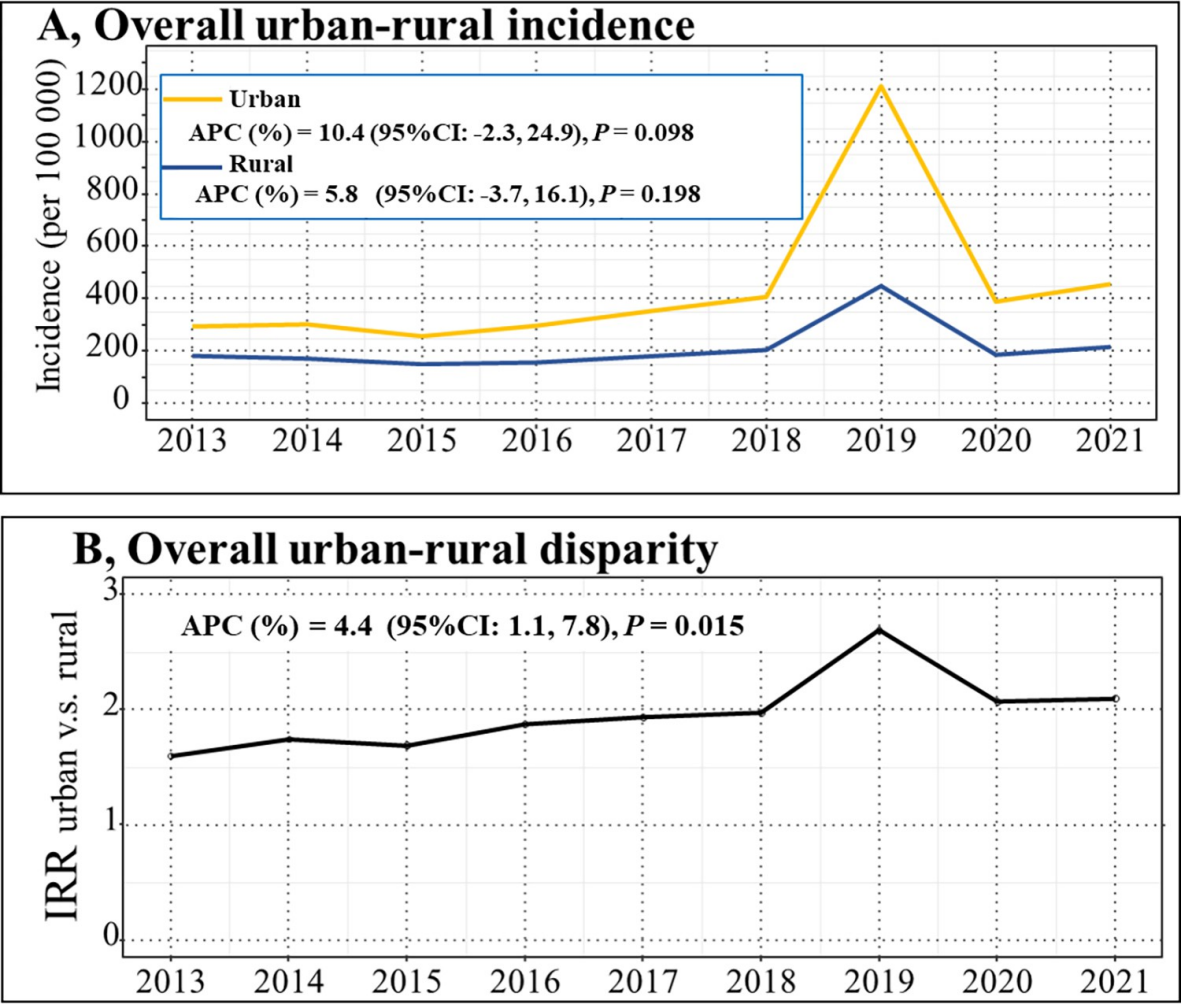
Trends in incidence, disparity of IRR, and APC of notifiable infectious diseases among children, adolescents, and youths in urban and rural areas from 2013 to 2021. ** Note:** Overall incidence for total infectious disease between urban and rural (subfigure A), and overall urban–rural disparity for IRR and its APC change from 2013 to 2021 (subfigure B). The APC for incidence was calculated using the yearly incidence rates from 2013 to 2021. Similarly, the APC for the IRR was determined based on the annual IRR figures from 2013 to 2021. APC, annual percent change; IRR, incidence rate ratio.

One notable occurrence was the outbreak of seasonal influenza in 2019 that affected children, adolescents, and youths in both urban and rural areas, so we conducted the analysis before and after 2019, excluding seasonal influenza. Results before excluding seasonal influenza showed that the incidence of infectious diseases increased from 293 and 183 per 100,000 in 2013 to 1,212 and 450 per 100,000 in 2019 among urban and rural populations, respectively (**S2 Table**), demonstrating an APC of 10.4% (95% CI [−2.3, 24.9]; *p* = 0.098) and 5.8% (95% CI [−3.7, 16.1]; *p* = 0.198) for these 2 groups (**Fig 1A**). This increase was primarily driven by pertussis, seasonal influenza, infectious diarrhea, gonorrhea, and syphilis in both urban and rural areas (**S7 Fig**). With the exception of the seasonal influenza, the incidence of infectious diseases remained stable from 276 and 176 per 100,000 in 2013 to 269 and 142 per 100,000 in 2021 among urban and rural populations, respectively (**S2 Table**). The overall disparity between urban and rural areas also grew each year, with the IRR increasing from 1.601 in 2013 to 2.098 in 2021 (**S2 Table**), indicating an APC of 4.4% (95% CI: [1.1, 7.8]; *p* = 0.015) (**Fig 1B**). Notably, the largest urban–rural disparity was observed during the outbreak of seasonal influenza in 2019, with an IRR of 2.695 (95% CI [2.688, 2.702]; *p* < 0.001) (**S2 Table**).

### Urban–rural disparity by category

**Fig 2:**
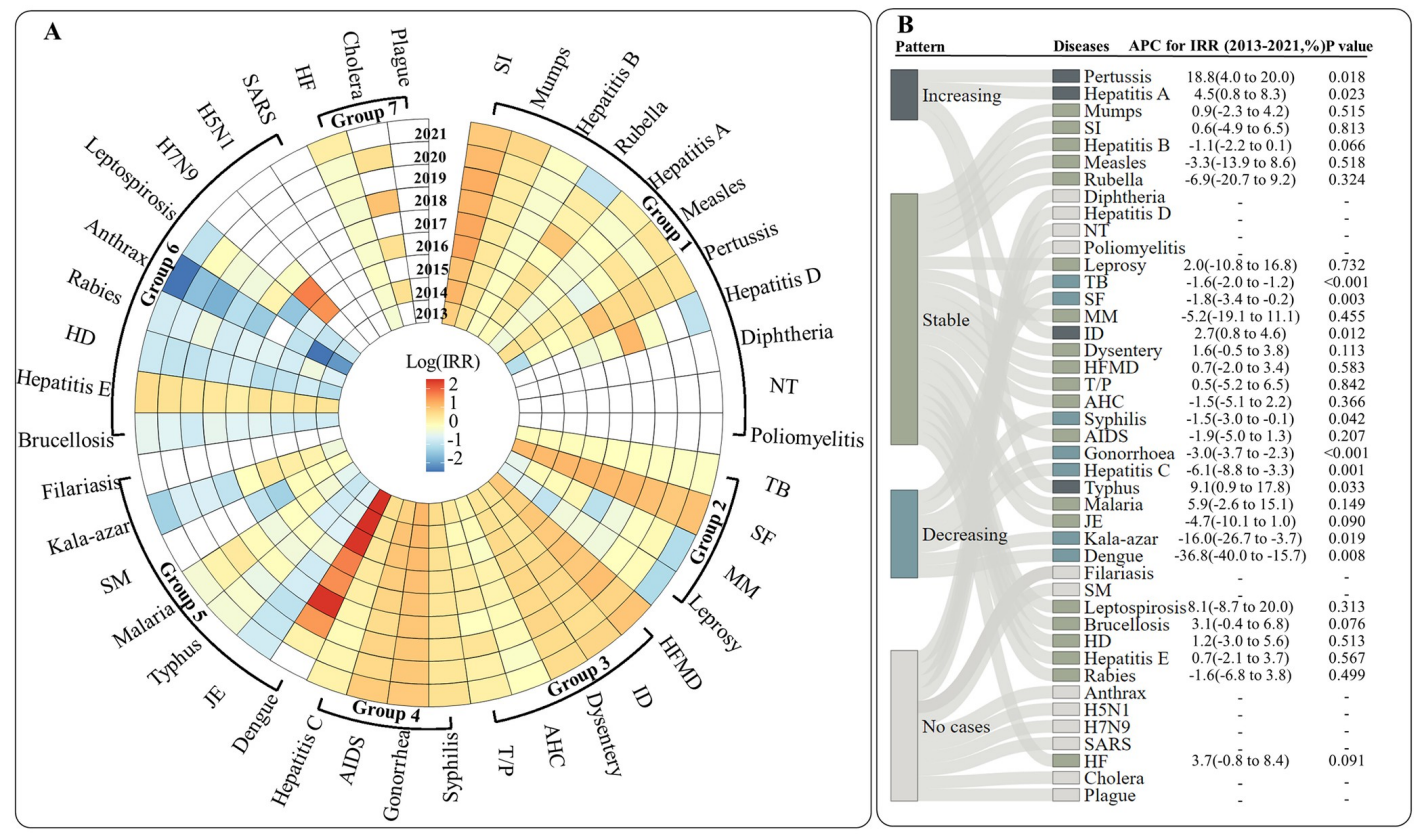
Patterns in incidence disparity of log transferred incidence rate ratio (log-IRR) and the change patterns by each specific infectious disease and years among Chinese children, adolescents, and youth from 2013 to 2021. **Note:** The urban–rural disparity of log transferred incidence rate ratio (log-IRR) (subfigure A), and their change patterns based on the APC (subfigure B). The uppercase letters A–G in the subfigure A represent the 7 categories of infectious disease: Group 1, vaccine preventable; Group 2, bacteria; Group 3, gastrointestil and enterovirus; Group 4, sexually transmitted and bloodborne; Group 5, vectorborne; Group 6, zoonotic; Group 7, quarantinable. The forest plot in the subfigure B represent the APC for IRR, and the actual number of APC (95% CI) and the *p* value are also represented in the subfigure B. SI, seasonal influenza; NT, neonatal tetanus; TB, tuberculosis; SF, scarlet fever; MM, meningococcal meningitis; T/P, typhoid fever and paratyphoid fever; HFMD, hand, foot, and mouth disease; AHC, acute hemorrhagic conjunctivitis; ID, infectious diarrhea; AIDS, acquired immune deficiency syndrome; SM, schistosomiasis; JE, Japanese encephalitis; HD, hydatid disease; SARS, severe acute respiratory syndrome; HF, hemorrhagic fever; IRR, incidence rate ratio; APC, annual percent change.

The urban–rural disparity by category and specific diseases over years, and most notifiable infectious diseases in both urban and rural areas presented a decreased or stable pattern in the incidence during the 9 years, but only several specific diseases witnessed an increased pattern in the incidence (**S7 Fig**), resulting in an overall increased pattern for both urban and rural diseases burdens and their widened disparity, such as pertussis, hepatitis A, infectious diarrhea, and typhus (**Fig 2**). Both males and females show a similar urban–rural disparity in each specific infectious disease by year (**S3 Table** and **S8 Fig**).

#### Vaccine preventable

The outbreak of seasonal influenza in 2019 drove an overall increase in the incidence of vaccine preventable diseases. Furthermore, the incidence of vaccine preventable diseases among children and adolescents increased slightly from 2013 to 2021 in both urban areas (133 to 225 per 100,000) and in rural areas (91 to 100 per 100,000), without significant trend (**S3 Table**). This resulted in an increasing urban–rural disparity with the IRR rising from 1.472 in 2013 to 2.246 in 2021 (APC = 7.5%; 95% CI [3.5, 11.7]; *p* = 0.003) during the period (**S3 Table and S5A and S6A Figs**). The long-term patterns of 11 vaccine preventable diseases in both urban and rural areas were consistent (**S7 Fig**), with higher incidence in urban areas than in rural areas, and the disparity continued to widen (**Figs 2 and S6A**). However, the patterns of specific diseases varied. Pertussis and seasonal influenza incidence increased, hepatitis A, hepatitis B, and measles incidence decreased, while mumps incidence remained stable (**S7 Fig**). However, it is noteworthy that the confidence interval for rubella is considerably wide in both urban and rural areas, which implies a considerable degree of uncertainty.

#### Bacteria

The incidence of bacterial infectious diseases in both urban and rural areas remained stable between 2013 and 2019 (**S5B Fig**), with urban areas having a higher incidence than rural areas (**S6B Fig**). However, a significant decrease was observed in 2020 and 2021, with decreasing APCs of −4.9 (95% CI [−10.5, 1.0]; *p* = 0.087) and −3.3 (95% CI [−6.5, 0.1]; *p* = 0.056) from 2013 to 2021 for urban and rural areas, respectively (**S5B and S6B Figs**). Over the period of 2013 to 2021, the urban–rural disparity in bacterial infectious diseases remained stable between 2013 and 2019, with the IRR ranging between 1.2 and 1.4, but significantly decreased in 2020 (IRR = 1.045; 95% CI [1.031, 1.060]; *p* < 0.001) (**S3 Table**). Among the 4 types of bacterial infections, tuberculosis and scarlet fever consistently had a higher incidence in urban areas than in rural areas (**Fig 2A**), but the urban–rural disparity has gradually decreased (**Fig 2B**).

#### Gastrointestinal and enterovirus

The incidence of gastrointestinal and enteroviral diseases has shown a sustained upward pattern in both urban and rural areas, increasing from 91 to 153 cases per 100,000 in urban areas and from 46 to 67 cases per 100,000 in rural areas from 2013 to 2021 (**S3 Table**). Throughout this period, there has been a consistent and substantial 2-fold urban–rural disparity (**S5C and S6C Figs**). Among the 5 diseases that fall under this category, all have demonstrated long-term, stable urban–rural disparities, except for infectious diarrhea, which has seen a slight increase in such disparities (**Fig 2**). While the incidence of infectious diarrhea has continued to rise in both urban and rural areas, both hand, foot, and mouth disease (HFMD) and acute hemorrhagic conjunctivitis have remained stable, and typhoid fever, paratyphoid fever, and dysentery have all shown a decreasing pattern over this period (2013 to 2021) (**S7 Fig**).

#### Sexually transmitted and bloodborne

The incidence of sexually transmitted and bloodborne diseases in both urban and rural populations increased significantly from 2013 to 2021, from 25 to 47 cases per 100,000 (urban) and from 12 to 23 cases per 100,000 (rural) (**S3 Table**), with a substantial annual percentage increase of 6.4% (95% CI [4.0, 8.9]; *p* < 0.001) in urban areas and 8.0% (95% CI [6.3, 9.7]; *p* < 0.001) in rural areas (**S5D and S6D Figs**). Urban areas exhibited about twice the incidence of rural areas, and the urban–rural disparity in the incidence of sexually transmitted and bloodborne diseases remained consistent, with an IRR ranging from 1.976 to 2.218 (**S3 Table**). Moreover, the incidence of both gonorrhea and syphilis rose, particularly in rural areas, while HIV/AIDS remained stable over the long term, and hepatitis C declined (**Figs 2** and **S7**).

#### Vectorborne

Vector-borne diseases have been generally prevalent at a low level, except for an outbreak for dengue in urban areas in 2014. The incidence of these diseases has shown a decreasing pattern in rural populations, dropping from 0.5 in 2013 to 0.1 per 100,000 in 2021 (**S3 Table**), with APCs of −12.0% (95% CI [−21.5, −1.4]; *p* = 0.033) (**S5E Fig**). Conversely, the decreasing pattern observed in urban areas did not reach statistical significance, as indicated by APCs of −25.7% (95% CI [−45.3, 1.0]; *p* = 0.056) (**S5E Fig**). This overall downward pattern was driven by the decline of each individual disease, including malaria, kala-azar (visceral leishmaniasis), and Japanese encephalitis in both urban and rural areas (**S7 Fig**). As a result, the urban–rural disparity in dengue incidence decreased notably, from an IRR of 35.908 to 1.300 (**Fig 2**). Japanese encephalitis and typhus consistently showed higher incidence in rural than urban areas, while malaria alternated between the 2 areas (**S7 Fig**). The overall downward pattern also drove a narrowing urban–rural disparity in vectorborne infectious diseases, from 1.649 in 2013 to 0.687 in 2021, and the urban–rural disparity in the incidence of vector-borne infectious diseases reversed in 2020 (**S3 Table**).

#### Zoonotic

The incidence of zoonotic infectious diseases was consistently higher in rural than urban areas, with rates of 1.1 and 0.7 per 100,000, respectively (**Table 1**), resulting in a consistent and large urban–rural disparity with IRR ranging from 0.578 to 0.729 during the period (2013 to 2021) (**S3 Table**). The APC in urban and rural areas were −1.0% (95% CI [−5.0, 3.2]; *p* = 0.585) and −2.6% (95% CI [−7.3, 2.4]; *p* = 0.251), respectively (**S5F** and **S6F Figs**). The incidence of hepatitis E, leptospirosis, and rabies decreased in both urban and rural areas, while the incidence of hydatid disease, anthrax, and brucellosis remained stable (**S7 Fig**). Excluding single digit cases of H7N9, only hepatitis E showed a higher incidence in urban areas with an IRR of 1.680, while other zoonotic diseases dominated in rural areas (**Fig 2**).

#### Quarantinable

The incidence of quarantinable diseases exhibited a fluctuating pattern over time, with APCs of 1.6% (95% CI [−5.7, 9.4]; *p* = 0.632) and 2.1% (95% CI [−7.0, 3.0]; *p* = 0.355) in urban and rural areas, from 2013 to 2021, respectively (**S5G and S6G Figs**). Hemorrhagic fever, being the main quarantinable disease, remained stable in both urban and rural areas during the nine-year study period (**S7 Fig**), resulting in a higher incidence in rural areas than in urban areas, and a consistent urban–rural disparity from 2013 to 2020 with an IRR ranging from 0.8 to 0.9 (**Fig 2 and S3 Table**). However, the disparity increased notably in urban areas, resulting in the IRR reversing to 1.422 (95% CI [1.229, 1.646]; *p* < 0.001) in 2021 (**S3 Table**).

### Urban–rural disparity by region

**Fig 3:**
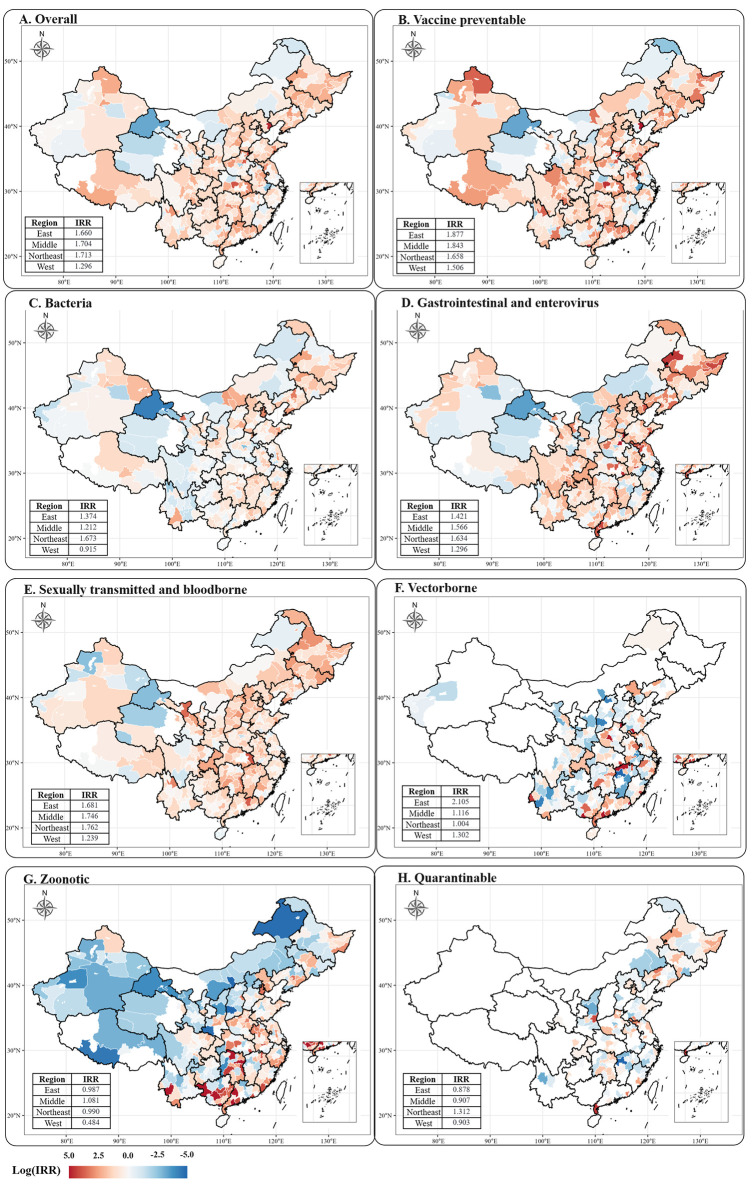
Regional disparity in log transferred incidence rate ratio (log-IRR) among Chinese children, adolescents, and youth from 2013 to 2021. **Notes:** Subfigure A to H showed the regional disparity of total and specific infectious disease category using the index of log transferred incidence rate ratio (log-IRR) for children, adolescents, and youth aged 4 to 24 years. The base map was obtained from Natural Earth (https://naturalearthdata.com). IRR, incidence rate ratio.

**Fig 3** presents a graphical representation of the regional distribution of infectious diseases in both urban and rural areas, revealing various degrees of geographic disparities. The regional incidence distribution of infectious diseases in rural and urban areas was generally comparable, with the highest incidence rates in the eastern regions and the lowest in the northeastern regions (**S4 Table and S9A and S10 Figs**). In the eastern regions, the average incidence rates in rural and urban areas were 467 and 282 per 100,000, respectively, with an IRR of 1.66 (**Fig 3A and S4 Table**). In the northeastern regions, the infection rates in urban and rural areas were 229 and 134 per 100,000, respectively, with the largest IRR of 1.71 (**Fig 3A and S4 Table**). Conversely, the western regions had the lowest urban–rural disparities, with a lowest IRR of 1.30 (**Fig 3A and S4 Table**).

The geographic distribution of urban–rural disparities in vaccine preventable diseases, bacterial infections, gastrointestinal and enterovirus infections, sexually transmitted, and bloodborne diseases were similar to the overall distribution of infectious diseases, with the lowest IRR in western regions (**Figs 3B, 3C, 3D, 3E, S9, and S10**). Interestingly, bacterial infections even had a reversal in urban–rural disparities in western regions, with an IRR of 0.915 (**Figs 3C and S9C and S4 Table**). Vectorborne diseases had a significant urban–rural disparity in eastern regions, with the largest IRR of 2.105 (**Figs 3G and S9G and S4 Table**). On the other hand, the urban–rural disparities in zoonotic diseases were the lowest in western regions, with a notable IRR of 0.484, indicating that the rural western population had a higher incidence of zoonotic diseases, whereas the middle regions had a higher incidence in urban areas, with IRRs of 1.081 (**Figs 3F and S9G and S4 Table**). As for quarantinable infectious diseases, urban areas had higher incidence rates than rural areas in northeastern regions (IRR: 1.312), while in other regions, rural areas had higher incidence rates than urban areas (**Figs 3H and S9H and S4 Table**).

Socioeconomic factors, including gross domestic product (GDP) and urbanization, are linked to infectious disease rates, as shown in the **S11 and S12 Figs**. As these factors improve, a U-shaped relationship with disease incidence is observed, with variations among different disease categories (**S11 and S12 Figs**). **S13 Fig** displays the inequality in notifiable infectious diseases among Chinese children, adolescents, and youth from 2013 to 2021 in the district/county level. During the nine-year period, overall incidence of infectious diseases was unequally distributed, with Gini coefficient of 0.592 (calculated based on the ranked GDP) and 0.616 (calculated based on the ranked urbanization). Based on the urbanization, vaccine preventable diseases also showed the greatest disparity (GINI: 0.726), followed by gastrointestinal and enterovirus diseases (GINI: 0.624) (**S13B Fig**). More detailed results on inequality can be found in **S2 Text and S14 and S15 Figs**.

## Discussion

To our knowledge, this is the first study to use a nationwide dataset of over 8.44 million cases to evaluate the long-term variations of urban–rural disparities and identify specific diseases driving these discrepancies among children, adolescents, and youths aged 4 to 24 years in China. Over the nine-year period, while the overall incidence of infectious diseases increased in both urban and rural areas due to major diseases like pertussis, seasonal influenza, infectious diarrhea, gonorrhea, and syphilis, most notifiable disease categories either decreased or remained stable. However, the burden of infectious diseases in urban populations was more than double than in rural areas, with this gap widening over time. Specific diseases, such as pertussis and hepatitis A among vaccine-preventable diseases, tuberculosis and scarlet fever among bacterial diseases, infectious diarrhea among gastrointestinal and enterovirus diseases, typhus among vectorborne diseases, and 4 sexually transmitted and bloodborne diseases, predominantly drove this disparity. Yet, this increased disparity was offset by most bacterial diseases, zoonotic diseases, and quarantinable diseases represented by hemorrhagic fever, as well as most vectorborne diseases. Geographically, the disparity was generally consistent, with noticeable high urban–rural disparities in zoonotic diseases in eastern China but a reversed disparity in western regions. Moreover, socioeconomic factors, including GDP and urbanization, were linked to infectious disease rates with a U-shaped relationship between them. Our study provides valuable insights into understanding infectious disease disparities between urban and rural populations and offers crucial guidance for addressing these inequalities in the future.

The incidence of infectious diseases among Chinese children and adolescents showed a slight upward trend from 2013 to 2021; meanwhile, a small number of infectious diseases have driven higher burdens in urban areas, widening urban–rural disparities. While a previous study in China covering the period from 2008 to 2015 indicated a decline in the incidence of most notifiable infectious diseases among Chinese children and adolescents [26], our current investigation reveals a gradual increase since 2015, predominantly in urban areas. With approximately half of Asia’s population currently residing in urban areas, and an expected increase to 64% by 2050 [35], urban regions are likely to become the primary epicenter for infectious diseases transmission in the future. In the process of rapid urbanization, the high population density and close proximity of individuals in cities create ideal conditions for the spread of infectious diseases, particularly respiratory infections, as demonstrated by the ongoing COVID-19 pandemic [3]. Since the onset of the COVID-19 pandemic, the widespread implementation of strict non-pharmaceutical interventions has significantly reduced the occurrence rates of nearly all infectious diseases globally [36]. Notably, respiratory and gastrointestinal infections have experienced particularly notable reductions [36]. Nevertheless, the implemented policies have contributed to a resurgence in respiratory system diseases. Over time, it is expected that the incidence rates of diverse infectious diseases will gradually approach historical levels, possibly resulting in a renewed surge of outbreaks attributed to waning immunity [37].

The larger urban–rural disparity in infectious diseases may benefit from ongoing improvements in urban surveillance systems. Continuous enhancements in monitoring systems, expanded coverage, and improved detection technology have led to increased identification of legally mandated infectious diseases in urban environments [29,38]. Higher education has led to increased awareness and willingness to actively accept relevant infectious disease detection [39]. On the other hand, rural areas face challenges such as limited access to medical care after infection and low health literacy, relative to urban areas [40]. Despite superior healthcare resources in urban areas, higher infectious disease rates persist compared to rural areas, likely due to elevated population density and confined public spaces [41,42]. The growing urban population further exacerbates disparities in healthcare resource accessibility within cities, contributing to intra-city healthcare resource inequality [43].

We have discovered a positive trend in the prevention and control of most vaccine-preventable diseases, with a continuing decline in their incidence. However, 2 major diseases, pertussis and hepatitis A, have shown a disproportionate increase in urban areas compared to rural areas, significantly widening overall urban–rural disparities. This trend underscores the imperative of ensuring equitable vaccine coverage, especially in low- and middle-income countries (LIMICs) undergoing rapid urbanization and economic growth [44,45]. Despite China’s 2008 initiative to enhance immunization coverage and broaden vaccine availability, challenges persist, particularly with seasonal influenza, which falls outside the immunization program and requires payment. Pertussis, despite effective controlled since planned immunization in 1978, demands vaccination before age 2 due to waning protective effects. Countries with high vaccination rates for diphtheria, tetanus, and pertussis, such as the United States and Australia, have observed pertussis resurgence after years of low incidence rates [46–48]. Our research in China, where pertussis vaccine coverage exceeds 99%, has identified a similar phenomenon [49,50]. Exploring urban–rural differences in vaccine-preventable infectious diseases among children and adolescents in China can help promote vaccine accessibility and coverage equity, particularly given China’s rapid but uneven urbanization development. This research is also relevant for LMICs worldwide that face limited healthcare infrastructure and lack access to vaccines, posing a serious public health concern [51].

In terms of bacterial diseases, tuberculosis remains prevalent in both urban and rural China. While our study indicates a decrease in tuberculosis incidence among children and adolescents in China, it still remains a leading cause of death in this age group [52], highlighting a significant urban–rural disparity in bacterial disease burden. Despite the declining trend observed in tuberculosis cases, as well as consistent urban–rural differences, our findings underscore China’s progress in tuberculosis prevention and control efforts. However, the challenge of less access to care in rural areas remains, and our study found that urban–rural disparities are particularly pronounced in the west, and that the urban–rural divide that drives bacterial disease is reversing, making rural areas a priority for tuberculosis intervention efforts aimed at reducing mortality [53]. Previous study reported that China’s center and western regions showed a greater decline in adult tuberculosis incidence compared to the western regions [54]. Similar to the disparity in childhood tuberculosis, adult tuberculosis occurs more frequently in rural than urban locations in western regions [55–57], where around 71% of tuberculosis patients reside [54,58]. It is worth noting that the incidence of tuberculosis among children and adolescents is similar in both urban and rural areas, indicating a significant increase in incidence of tuberculosis between this group and adulthood. As a result, children and adolescents in rural areas play a critical role in tuberculosis elimination efforts. In terms of scarlet fever, urban areas have a rate nearly 3 times higher than rural areas, with children and adolescents being the most affected group. Although previous studies have reported an increase in scarlet fever incidence among this age group in China [26], as well as resurgences in other regions [59], this study did not find such an increase, which could be due to its cyclical pattern of ups and downs [60]. However, the high incidence of scarlet fever in urban areas remains a concern even during periods of decline. Therefore, age- and region-specific control measures are needed to address the variations of urban–rural disparities in bacterial diseases.

Sexually transmitted and bloodborne diseases, particularly for sexually transmitted diseases (STDs), pose a significant public health challenge, especially in urban areas where their incidence is consistently higher than in rural areas. This disparity is most prominent in areas with lower socioeconomic levels, but as the economy improves, the gap tends to decrease. Nonetheless, the increasing trend in the incidence of these diseases in both urban and rural areas is a cause for concern, presenting a major challenge for countries worldwide [26,61,62]. It is important to note that the tuberculosis is closely related to STDs [63]. Together, tuberculosis and STDs caused 1.5 million deaths globally in 2020, including 241,000 individuals who were HIV–positive [64]. Given the co-occurrence of these diseases, there is a growing recognition of the need to consider both in the prevention and control of infectious diseases among children and adolescents. While the incidence of STDs is most pronounced in urban areas, there is an increasing risk of these diseases spreading to rural areas. This trend highlights the essential to implement comprehensive prevention and control strategies to mitigate the urban–rural disparity in STDs. This includes improving access to education and prevention measures, such as promoting sexual health awareness and providing free or low-cost condoms in both urban and rural areas. Additionally, strengthening healthcare systems by expanding STDs testing and treatment services, particularly in underserved rural areas, can help reduce the incidence and spread of STDs and bridge the urban–rural gap in disease burden.

The incidence of gastrointestinal and enterovirus diseases is significantly higher in urban areas, being 2.1 times higher than in rural areas. HFMD and infectious diarrhea are the main gastrointestinal and enterovirus infectious diseases. While the urban–rural disparity in HFMD aligns with previous research [65], the difference in mortality and severe cases between urban and rural areas differs from the incidence gap [65]. Rural areas exhibit lower incidence rates but higher mortality and severe case rates compared to urban areas [65]. Moreover, HFMD recurrence is more prevalent in urban areas than in rural ones, underscoring the disproportionate burden on urban regions [66]. Strengthening symptom monitoring and warning systems in both urban and rural areas is essential to address this issue.

Furthermore, our study offers a comprehensive overview of the urban–rural disparity in the incidence and patterns of vector-borne, zoonotic, and quarantinable infectious diseases in China. While the overall trend for vector-borne diseases is declining, driven by dengue reduction, the urban–rural gap is narrowing. Zoonotic diseases consistently exhibit higher incidence rates in rural areas, indicating a notable urban–rural disparity. Quarantinable diseases demonstrate a fluctuating pattern with a persistent urban–rural gap, albeit with a reversal in urban areas in 2021. These findings underscore the importance of addressing urban–rural disparities in infectious diseases, particularly for zoonotic diseases. Efforts should focus on enhancing rural healthcare infrastructure and access to medical services to improve disease detection and treatment. Additionally, implementing targeted public health campaigns in rural areas can raise awareness about disease prevention and control measures, ultimately reducing the gap between urban and rural areas.

The study has several strengths. In this investigation of infectious diseases, we utilized a robust and large sample surveillance system with over 8.44 million cases, the CISDCP, which systematically and representatively monitors infectious diseases and covers over 85% of all health facilities in China in both urban and rural areas [26,28]. Moreover, the study included all children and adolescents, and youths whether or not they were attending school, making it the most comprehensive representation of the epidemiological situation of infectious diseases in China. The study’s strength lies in the high accuracy of diagnosis for all infectious diseases. Although CISDCP recorded suspected cases, carriers of pathogens, and confirmed cases, only patients with diagnoses supported by uniform clinical standards and laboratory tests were included in this investigation.

However, several potential limitations should be noted. Firstly, while the study utilized a robust surveillance system, it is possible that some cases were missed due to underreporting or insufficient surveillance in certain areas. Furthermore, the population data used to calculate incidence rates was based on the annual average population of each region, which may not account for population movement and could introduce errors. Nevertheless, it is worth noting that since the majority of children and adolescents are students with relatively low mobility, this limitation may be somewhat mitigated. Despite the fact that the CDCs conducts data audits on infectious diseases in the reporting system, differences in the professional levels of various CDCs may introduce errors, potentially leading to an underestimation of the incidence rates of infectious diseases in certain regions. However, it is noteworthy that the same case undergoes reviews at 3 different levels: country, city, and provincial CDCs during the submission process. This procedural scrutiny is anticipated to, to a certain extent, alleviate the prospect of underestimating infectious disease incidence rates attributable to divergences in CDC expertise at varying levels. We classified long-term patterns using APC values and *P* values. It is crucial to note that a nonsignificant *P* value does not necessarily mean the absence of a pattern; a pattern may still be present. When interpreting results, caution is needed with *P* values due to potential influences from factors like sample size and variability. The study’s participants were aged 4 to 24 years, showing different infectious disease patterns from the 0 to 4 age group. Specifically, gastrointestinal infections are more prevalent in the younger group, while sexually transmitted and zoonotic diseases are less common [67]. Therefore, the study’s findings, particularly regarding urban–rural disparities in gastrointestinal infections, may not apply to the 0 to 4 age group and could underestimate these disparities. Finally, the study only considered legally notified infectious diseases, which may not capture all infectious diseases in this age group. Despite these limitations, this study represents a critical step towards developing targeted public health interventions to reduce the burden and bridge the urban–rural disparity of infectious diseases among children and adolescents, and youths in China.

In summary, as urbanization accelerates, the urban–rural disparities in the spectrum of 43 infectious diseases among Chinese children and adolescents are becoming increasingly complex. While progress has been made in controlling most infectious diseases in China, our findings indicate rising disease incidence rates in both urban and rural areas, primarily attributed to increases in seasonal influenza and several other diseases including pertussis, infectious diarrhea, gonorrhea, and syphilis. Importantly, the burden of infectious diseases among urban populations exceeds that of rural areas by over 2-fold, with disparities widening over time. This disparity was driven mainly by several specific diseases, such as pertussis and seasonal influenza among vaccine-preventable diseases, tuberculosis and scarlet fever among bacterial diseases, infectious diarrhea among gastrointestinal and enterovirus diseases, typhus among vectorborne diseases, and 4 sexually transmitted and bloodborne diseases. Therefore, future efforts while maintaining existing achievements should focus on developing targeted interventions to reduce the burden of these diseases and address these disparities. Targeted interventions must focus on strengthening healthcare infrastructure and access in rural areas, as well as implementing comprehensive public health campaigns to raise awareness about disease prevention and control measures. Additionally, improving surveillance systems and enhancing early detection and treatment services can help mitigate the spread of infectious diseases, particularly in underserved rural communities. In tackling STDs and vaccine-preventable diseases, prioritizing education, preventive measures, and healthcare services, along with enhancing vaccine accessibility and coverage, particularly in urban centers, is vital to narrowing the urban–rural disparity in disease burden among children and adolescents.

## Supporting information

S1 Gather Checklist Checklist of information that should be included in new reports of global health estimate. (PDF)

S1 Text Analysis plan. (DOCX)

S2 Text Results of inequality in infectious diseases. (DOCX)

S1 Methods Additional methods for association between socioeconomic index and incidence. (DOCX)

S2 Methods Lorenz curve and Gini coefficient. (DOCX)

S1 Table The incidence of total 43 notifiable infectious diseases by category and specific diseases by sex and urban and rural. Note: *Total numbers from 2013 to 2021; # Average incidence 2013 to 2021; -, no cases. SI, seasonal influenza; NT, neonatal tetanus; TB, tuberculosis; SF, scarlet fever; MM, meningococcal meningitis; T/P, typhoid fever and paratyphoid fever; HFMD, hand, foot, and mouth disease; AHC, acute hemorrhagic conjunctivitis; ID, infectious diarrhea; AIDS, acquired immune deficiency syndrome; SM, schistosomiasis; JE, Japanese encephalitis; HD, hydatid disease; SARS, severe acute respiratory syndrome; HF, hemorrhagic fever.
(DOCX)

S2 Table Trends in incidence and disparity of incidence rate ratio (IRR) of notifiable infectious diseases between urban and rural children, adolescents, and youth from 2013 to 2021. Note: IRR, incidence rate ratio.
(DOCX)

S3 Table Trends in incidence and disparity of incidence rate ratio (IRR) of notifiable infectious diseases by category among children, adolescents, and youths in urban and rural areas from 2013 to 2021. Note: IRR, incidence rate ratio.
(DOCX)

S4 Table The incidence and disparity of incidence rate ratio (IRR) of notifiable infectious diseases by category between children, adolescents, and youths in urban and rural areas in eastern, middle, northeastern, and western regions from 2013 to 2021. Note: IRR, incidence rate ratio.
(DOCX)

S1 Fig Vertical Reporting Structure, Chinese Infectious Diseases Reporting System (figure from China Information System for Disease Control and Prevention). Notes: CDC, Chinese Centers for Disease Control.
(TIF)

S2 Fig Distribution of urban and rural areas in China and the schematic diagram of the 3 levels of administrative division in China. Notes: Subfigure A presents the distribution of urban and rural areas in China; subfigure B presents the schematic diagram of the 3 levels of administrative division in China, which is based on the Sichuan province and Chengdu city. The base map was obtained from Natural Earth (https://naturalearthdata.com).
(TIF)

S3 Fig The incidence of 7 categories of notifiable infectious diseases by years from 2013 to 2021. (TIF)

S4 Fig The incidence of overall infectious diseases and 7 categories of notifiable infectious diseases, excluding seasonal influenza, by year from 2013 to 2021. Notes: Incidence calculations for specific infectious disease categories are based on excluding cases of seasonal influenza; subfigure A presents the incidence of overall infectious diseases excluding the seasonal influenza by urban and rural areas; subfigure B presents the total incidence by 7 categories from 2013 to 2021 in urban areas, while subfigure C presents the total incidence by 7 categories over the same period in rural areas.
(TIF)

S5 Fig Trends in incidence and annual percentage change (APC) of notifiable infectious diseases among children, adolescents, and youths in urban and rural areas by diseases category from 2013 to 2021. Notes: subfigure A–G presents the incidence of vaccine preventable, bacteria, gastrointestil and enterovirus, sexually transmitted and bloodborne, vectorborne, zoonotic, and quarantinable infectious diseases, respectively. The APC for the incidence in urban and rural areas is shown at the top of each subfigure.
(TIF)

S6 Fig Trends in disparity of incidence rate ratio (IRR) and annual percentage change (APC) of notifiable infectious diseases among children, adolescents, and youths in urban and rural areas, by diseases category from 2013 to 2021. Notes: Subfigure A–G presents the IRR of vaccine preventable, bacteria, gastrointestil and enterovirus, sexually transmitted and bloodborne, vectorborne, zoonotic, and quarantinable infectious diseases, respectively. The APC for the IRR is shown at the top of each subfigure.
(TIF)

S7 Fig Patterns in incidence in urban and rural, and the change patterns by each specific infectious disease and years among Chinese children, adolescents, and youth from 2013 to 2021. **Note:** Overall incidence changes for each specific infectious disease between urban and rural (subfigure A for urban and C for rural), and their change patterns based on the annual percentage change (APC) (subfigures B and D). The uppercase letters A–G in the subfigure A represent the 7 categories of infectious disease: Group 1, vaccine preventable; Group 2, bacteria; Group 3, gastrointestil and enterovirus; Group 4, sexually transmitted and bloodborne; Group 5, vectorborne; Group 6, zoonotic; Group 7, quarantinable. The forest plot in the subfigure B represent the APC for IRR, and the actual number of APC (95% CI) and the *p* value are also represented in the subfigure B. SI, seasonal influenza; NT, neonatal tetanus; TB, tuberculosis; SF, scarlet fever; MM, meningococcal meningitis; T/P, typhoid fever and paratyphoid fever; HFMD, hand, foot, and mouth disease; AHC, acute hemorrhagic conjunctivitis; ID, infectious diarrhea; AIDS, acquired immune deficiency syndrome; SM, schistosomiasis; JE, Japanese encephalitis; HD, hydatid disease; SARS, severe acute respiratory syndrome; HF, hemorrhagic fever; IRR, incidence rate ratio.
(TIF)

S8 Fig Patterns in incidence in urban and rural disparity of log transferred incidence rate ratio (log-IRR) in both males and females from 2013 to 2021. **Note:** Group 1, vaccine preventable; Group 2, bacteria; Group 3, gastrointestil and enterovirus; Group 4, sexually transmitted and bloodborne; Group 5, vectorborne; Group 6, zoonotic; Group 7, quarantinable. The forest plot in the subfigure B represent the APC for IRR, and the actual number of APC (95% CI) and the *p* value are also represented in the subfigure B. SI, seasonal influenza; NT, neonatal tetanus; TB, tuberculosis; SF, scarlet fever; MM, meningococcal meningitis; T/P, typhoid fever and paratyphoid fever; HFMD, hand, foot, and mouth disease; AHC, acute hemorrhagic conjunctivitis; ID, infectious diarrhea; AIDS, acquired immune deficiency syndrome; SM, schistosomiasis; JE, Japanese encephalitis; HD, hydatid disease; SARS, severe acute respiratory syndrome; HF, hemorrhagic fever; IRR, incidence rate ratio.
(TIF)

S9 Fig Regional distribution for urban and rural (subfigure A1–H1 for urban and subfigure A2–H2 for rural) of specific infectious disease category in incidence among Chinese children, adolescents, and youth. Notes: The uppercase letters A–G in the each panel represent the 7 categories of infectious disease: A, overall infectious disease; B, vaccine preventable; C, bacteria; DD, gastrointestil and enterovirus; E, sexually transmitted and bloodborne; F, vectorborne; G, zoonotic; H, quarantinable. Each panel included 2 figures, the map of incidence in urban and rural; regional distribution of total and specific infectious disease category in incidence was showed for those in urban (subfigure A1–H1) and rural (subfigure A2–H2). The base map was obtained from Natural Earth (https://naturalearthdata.com).
(TIF)

S10 Fig The regional classification of east, middle, northeast, and west in Chinese 31 provinces. Notes: The base map was obtained from Natural Earth (https://naturalearthdata.com).
(TIF)

S11 Fig The association between the infectious diseases and gross domestic product (GDP) in the district/county level. Notes: The log-transform was applied to the incidence and GDP. The blue line using the generalized additive models (GAMs) represents the nonlinear association of GDP and incidence of total and specific categorized notifiable infectious diseases. The dots present the district/county.
(TIF)

S12 Fig The association between the infectious diseases and urbanization in the district/county level. Notes: The log-transform was applied to the incidence and urbanization. The blue line using the generalized additive models (GAMs) represents the nonlinear association of urbanization and incidence of total and specific categorized notifiable infectious diseases. The dots present the district/county.
(TIF)

S13 Fig Lorenz curves and Gini coefficients of yearly incident case of infectious diseases among Chinese children, adolescents, and youth of total level from 2013 to 2021 (in the district/county level). Notes: The (dashed) diagonal line of equality depicted a theoretical scenario where infectious disease cases were equally distributed across the population. The Gini coefficients were calculated based on the Lorenz curves, with larger coefficients indicating more unequal distribution of infectious disease cases and smaller coefficients indicating a more equal distribution. In the subfigure A: The Lorenz curves were generated by sorting the GDP in each city/municipal from the lowest to the highest GDP. The x-axis and y-axis represented the cumulative percentage of the population ranked by the GDP and cumulative proportion of notifiable infectious diseases in the surveillance year. In the subfigure B: The Lorenz curves were generated by sorting the urbanization in each city/municipal from the lowest to the highest urbanization. The x-axis and y-axis represented the cumulative percentage of the population ranked by the urbanization and cumulative proportion of notifiable infectious diseases in the surveillance year.
(TIF)

S14 Fig Lorenz curves and Gini coefficients of yearly incident case of infectious diseases among Chinese children, adolescents, and youth of total level from 2013 to 2021 (in the city/municipal level). Notes: The (dashed) diagonal line of equality depicted a theoretical scenario where infectious disease cases were equally distributed across the population. The Gini coefficients were calculated based on the Lorenz curves, with larger coefficients indicating more unequal distribution of infectious disease cases and smaller coefficients indicating a more equal distribution. In the subfigure A: The Lorenz curves were generated by sorting the GDP per capita in each city/municipal from the lowest to the highest GDP per capita. The x-axis and y-axis represented the cumulative percentage of the population ranked by the GDP per capita and cumulative proportion of notifiable infectious diseases in the surveillance year. In the subfigure B: The Lorenz curves were generated by sorting the urbanization in each city/municipal from the lowest to the highest urbanization. The x-axis and y-axis represented the cumulative percentage of the population ranked by the urbanization and cumulative proportion of notifiable infectious diseases in the surveillance year.
(TIF)

S15 Fig Lorenz curves and Gini coefficients of yearly incident case of infectious diseases among Chinese children, adolescents, and youth of total level from 2013 to 2021 (in the district/county level) by regions. Notes: The (dashed) diagonal line of equality depicted a theoretical scenario where infectious disease cases were equally distributed across the population. The Gini coefficients were calculated based on the Lorenz curves, with larger coefficients indicating more unequal distribution of infectious disease cases and smaller coefficients indicating a more equal distribution. The Lorenz curves were generated by sorting the GDP in each city/municipal from the lowest to the highest GDP. The x-axis and y-axis represented the cumulative percentage of the population ranked by the GDP and cumulative proportion of notifiable infectious diseases in the surveillance year. Table for GINI coefficient shows the GINI coefficient.
(TIF)

## Abbreviations

APC: annual percent change
CISDCP: China Information System for Disease Control and Prevention
GDP: gross domestic product
HFMD: hand, foot, and mouth disease
IRB: Institutional Review Board
IRR: incidence rate ratio
SARS: severe acute respiratory syndrome
STD: sexually transmitted disease
