# Supplementary material for: Long-term variations of urban–Rural disparities in infectious disease burden of over 8.44 million children, adolescents, and youth in China from 2013 to 2021: An observational study
Source: PLoS Med. 2024 Apr 12;21(4):e1004374. doi: 10.1371/journal.pmed.1004374 (PMC11014433; doi:10.1371/journal.pmed.1004374)
Supplement: S1 Text — (DOCX) [file pmed.1004374.s002.docx]

# S1 Text. Analysis plan.

**Objective**

The primary objective of this study is to investigate the urban-rural disparity in the incidence of infectious diseases, examining trends from 2013 to 2021. Additionally, the study aims to assess the inequality between urban and rural areas concerning infectious diseases, encompassing seven disease categories and 43 individual infectious diseases.

**Hypothesis**

Overall Disparity Hypothesis: There is a general difference in the incidence rates of infectious diseases between urban and rural areas.

Category and Individual Disease Distribution Disparity Hypothesis: Different categories and individual infectious diseases exhibit significant variations in distribution between urban and rural areas.

Urban-Rural Heterogeneity Hypothesis: In both urban and rural regions, infectious diseases demonstrate heterogeneity, with greater internal heterogeneity observed in urban areas.

**Study participants**

Children and adolescents aged 4-24 years with notifiable infectious diseases

**Main outcomes**

Incidence of all-causes infectious diseases, seven disease categories and 43 individual infectious diseases.

**Stratification**

Region (Urban, rural)

GDP per capita: Stratum 1 (low income, ≤6278 US$ per year), Stratum 2 (lower middle income, 6278–7796 US$ per year), Stratum 3 (upper middle income, 7796–10794 US$ per year), and Stratum 4 (high income, >10794 US$ per year).

Urbanization: Stratum 1 (least urbanized, ≤50.00%), Stratum 2 (lower middle urbanized, 50.01–60.00%), Stratum 3 (upper middle urbanized, 60.01–70.00%), and Stratum 4 (most urbanized, >70.00%).

**Statistics**

Calculating the incidence by urban and rural areas:

$$incidence= {(Number of cases)}/{(Number of population)}$$

Calculating the urban-rural incidence rate ratio (IRR):

$$IRR={({incidence}_{urban})}/{({incidence}_{rural})}$$

Joinpoint regression models estimated annual percentage changes (APC) and subjected them to significance testing using a Z test.

To compare the inequality of urban and rural areas in infectious diseases, we utilized the Lorenz curve and its related Gini coefficient values.

**Analyses were actually performed**

In our initial analysis plan, we intended to use provincial per capita GDP for stratified analysis. However, during the actual analysis, we discovered that provincial-level economic indicators did not adequately reflect the economic disparities at the district/county level. Within the same province, there were significant variations in the economic status of different districts/counties. Therefore, we opted for a more detailed economic indicator in our actual analysis, namely the economic indicators at the district/county level. Nonetheless, due to the lack of specific district/county level per capita GDP data, we resorted to analyzing the total GDP and urbanization levels at the district/county level.

It is important to note that there is a significant amount of missing data for the GDP and urbanization at the district/county level (with GDP missing rate at 10% and urbanization missing rate at 64%). To assess the potential impact of these missing data on our study results, we implemented a supplementary measure, conducting a similar analysis with the city/municipal level per capita GDP and urbanization levels. This step is intended to provide a reference point to understand the implications of data missingness and to enhance the robustness of our research.
