# Supplementary material for: Long-term variations of urban–Rural disparities in infectious disease burden of over 8.44 million children, adolescents, and youth in China from 2013 to 2021: An observational study
Source: PLoS Med. 2024 Apr 12;21(4):e1004374. doi: 10.1371/journal.pmed.1004374 (PMC11014433; doi:10.1371/journal.pmed.1004374)
Supplement: S2 Text — (DOCX) [file pmed.1004374.s003.docx]

# S2 Text. Results of inequality in infectious diseases.

The relationship between the GDP, urbanization and the incidence of notifiable infectious diseases was shown in the **S11 Fig and S12 Fig**. As socioeconomic development improves, marked by higher GDP and urbanization levels, the U-shaped association between socioeconomic index and overall incidence of infectious diseases was observed. The association between socioeconomic level and infectious diseases varied by disease category (**S11 Fig and S12 Fig**).

**S13 Fig** displays the inequality in notifiable infectious diseases among Chinese children, adolescents, and youth from 2013 to 2021 in the district/county level. During the nine-year period, overall incidence of infectious diseases was unequally distributed, with Gini coefficient of 0.592 (calculated based on the ranked GDP) and 0.616 (calculated based on the ranked urbanization). The Lorenz curve illustrated inequality distribution of infectious diseases across the seven categories (**S13 Fig**). Notably, the vaccine preventable diseases showed the greatest disparity (GINI based on the GDP: 0.735). Based on the urbanization, vaccine preventable diseases also showed the greatest disparity (GINI: 0.726), followed by gastrointestinal and enterovirus diseases (GINI: 0.624), sexually transmitted and bloodborne diseases (GINI: 0.540), zoonotic (GINI: 0.521), vetorborne (GINI: 0.495), and quarantinable (GINI: 0.382) (**S13 Fig B**). The unequally distribution in the city/municipal level was observed, with Gini coefficient of 0.601 (calculated based on the ranked GDP per capita) and 0.587 (calculated based on the ranked urbanization) (**S14 Fig**). The Lorenz curve and GINI coefficient by regions also provided in the **S15 Fig.**
