# Supplementary material for: Long-term variations of urban–Rural disparities in infectious disease burden of over 8.44 million children, adolescents, and youth in China from 2013 to 2021: An observational study
Source: PLoS Med. 2024 Apr 12;21(4):e1004374. doi: 10.1371/journal.pmed.1004374 (PMC11014433; doi:10.1371/journal.pmed.1004374)
Supplement: S1 Methods — (DOCX) [file pmed.1004374.s004.docx]

**S1 Methods. Additional methods for association between socioeconomic index and incidence.**

To investigate the socioeconomic factors that may influence the incidence of infectious diseases, we collected two indicators - Gross Domestic Product (GDP) and urbanization - at the district/municipal levels for each year from 2013-2021, from the China statistical yearbook of the National and Provincial Bureau of Statistics of China. The yearbooks contain reliable data on real GDP for the major districts/municipals, allowing for a comparison of national macroeconomic growth adjusted for purchasing power parity exchange rates (expressed in US$ based on the exchange rate at the time of the survey). The urbanization ratio, which is calculated as the proportion of the population living in urban areas, serves as a crucial indicator of a region's economic level and social progress. It is worth noting that the cities and rural areas defined in this study are not the same as those used in the urbanization rate calculation.

To compare the inequality in infectious diseases, we utilized the Lorenz curve and its related Gini coefficient values. This approach allowed for the assessment of inequality, as well as the inequality in different infectious diseases. The Lorenz curve was generated by plotting the percentage of the proportion ranked by urbanization or GDP on the x-axis against the cumulative proportion of notified cases on the y-axis (more details in the S1 Methods). To depict a theoretical scenario of equal distribution of infectious diseases across the urbanization or GDP, the diagonal line of equality was plotted. Deviations of the Lorenz curve from this line indicate greater concentration of infectious diseases in certain areas. The Gini coefficient values, which range from 0 to 1 and correspond to the area between the line of equality and the Lorenz curve, serve as a measure of inequality, with higher values indicating greater inequality. Substantial missing data in district/county level GDP and urbanization (GDP missing rate at 10%, urbanization missing rate at 64%) led us to conduct a supplementary analysis using city/municipal level per capita GDP and urbanization to assess the impact on our study results. Generalized additive models (GAMs) were used to evaluate the non-linear associations of the GDP and urbanization and the incidence of total and specific categorized notifiable infectious diseases.
