# Supplementary material for: Long-term variations of urban–Rural disparities in infectious disease burden of over 8.44 million children, adolescents, and youth in China from 2013 to 2021: An observational study
Source: PLoS Med. 2024 Apr 12;21(4):e1004374. doi: 10.1371/journal.pmed.1004374 (PMC11014433; doi:10.1371/journal.pmed.1004374)
Supplement: S2 Methods — (DOCX) [file pmed.1004374.s005.docx]

# S2 Methods. Lorenz curve and Gini coefficient.

The process of plotting the Lorenz curve and calculating the Gini coefficient involves several key steps, based on the previous studies[1, 2]:

**1. Data Preparation**: Begin by collecting data on the number of infectious disease cases and the urbanization in each district.

**2. Data Sorting**: Sort the data in ascending order based on the urbanization. This step organizes the data for the subsequent calculations.

**3. Cumulative Proportions**: Calculate the percentage of the proportion ranked by urbanization at each data point as you progress through the sorted data. To do this, divide the rank of each data point by the total number of data points. This cumulative proportion represents a cumulative distribution function (CDF).

**4. Cumulative Cases**: Simultaneously, calculate the cumulative proportion of infectious disease cases at each data point as you move through the sorted data. This is done by summing the infectious disease cases as you progress through the sorted data. This cumulative proportion also forms a CDF, but for the variable of interest, which is the number of infectious disease cases.

**5. Lorenz Curve Plotting**: Create the Lorenz curve by plotting the percentage of the proportion ranked by urbanization on the x-axis and the cumulative proportion of infectious disease cases (CDF of cases) on the y-axis. Begin at the origin (0,0) on the graph because, at the outset, no proportion of the population has zero infectious disease cases. Proceed to connect each point representing the percentage of the proportion ranked by urbanization to the corresponding cumulative proportion of infectious disease cases as you move through the sorted data. This line constitutes the Lorenz curve.

**6. Line of Equality**: In addition to the Lorenz curve, plot a diagonal line from the origin (0,0) to the point (1,1). This diagonal line represents the line of equality, illustrating an ideal scenario where infectious diseases are evenly distributed across the entire population.

**7. Gini Coefficient Calculation**: Calculate the Gini coefficient by determining the area between the Lorenz curve and the line of equality (denoted as A) and dividing it by the total area under the line of equality (including the area of perfect equality, denoted as B).

Gini = (A) / (A + B)

**8. Interpretation**: The Gini coefficient is a value between 0 and 1. A Gini coefficient of 0 signifies perfect equality, indicating that all individuals have an equal share of the variable, such as infectious disease cases. Conversely, a Gini coefficient of 1 represents perfect inequality, where one individual or group holds the entirety of the variable. The Gini coefficient closer to 1 indicates greater inequality in the distribution of the variable, which, in this context, relates to infectious disease cases.

In summary, the Lorenz curve visually represents the distribution of infectious disease cases across the population, while the Gini coefficient quantifies the level of inequality in this distribution. The Gini coefficient serves as a valuable measure for assessing disparities, with higher values indicating greater inequality in the distribution of infectious disease cases among different districts or areas.

For GDP, we use the same method to plot Lorenz curve.

**Reference:**

1. Mody A, Pfeifauf K, Geng EH. Using Lorenz Curves to Measure Racial Inequities in COVID-19 Testing. JAMA Netw Open. 2021;4(1):e2032696. Epub 2021/01/09. doi: 10.1001/jamanetworkopen.2020.32696. PubMed PMID: 33416882; PubMed Central PMCID: PMCPMC7794664.

2. Darkwah KA, Nortey EN, Lotsi A. Estimation of the Gini coefficient for the lognormal distribution of income using the Lorenz curve. Springerplus. 2016;5(1):1196. Epub 2016/08/16. doi: 10.1186/s40064-016-2868-z. PubMed PMID: 27516934; PubMed Central PMCID: PMCPMC4963343.
