# Supplementary material for: Long-term variations of urban–Rural disparities in infectious disease burden of over 8.44 million children, adolescents, and youth in China from 2013 to 2021: An observational study
Source: PLoS Med. 2024 Apr 12;21(4):e1004374. doi: 10.1371/journal.pmed.1004374 (PMC11014433; doi:10.1371/journal.pmed.1004374)
Supplement: S1 Table — Note: *Total numbers from 2013 to 2021; # Average incidence 2013 to 2021; -, no cases. SI, seasonal influenza; NT, neonatal tetanus; TB, tuberculosis; SF, scarlet fever; MM, meningococcal meningitis; T/P, typhoid fever and paratyphoid fever; HFMD, hand, foot, and mouth disease; AHC, acute hemorrhagic conjunctivitis; ID, infectious diarrhea; AIDS, acquired immune deficiency syndrome; SM, schistosomiasis; JE, Japanese encephalitis; HD, hydatid disease; SARS, severe acute respiratory syndrome; HF, hemorrhagic fever. (DOCX) [file pmed.1004374.s006.docx]

**S1 Table**. The incidence of total 43 notifiable infectious diseases by category and specific diseases by sex and urban and rural.

| **Category** | **Rural** | | | | | |  | **Urban** | | | | | |
| --- | --- | --- | --- | --- | --- | --- | --- | --- | --- | --- | --- | --- | --- |
|  | **Male** | | | **Female** | | |  | **Male** | | | **Female** | | |
|  | **Cases (n)*** | **Yearly incidence** # | **Age range** | **Cases (n)*** | **Yearly incidence** # | **Age range** |  | **Cases (n)*** | **Yearly incidence** # | **Age range** | **Cases (n)*** | **Yearly incidence** # | **Age range** |
| **Overall** | **2302359** | **215.626** | **-** | **1653554** | **206.251** | **-** |  | **2611937** | **452.897** | **-** | **1875106** | **422.85** | **-** |
| **Vaccine preventable** | **1150686** | **108.001** | **-** | **851854** | **106.758** | **-** |  | **1423726** | **247.7** | **-** | **1083096** | **245.785** | **-** |
| SI | 553046 | 52.502 | 5-23 | 440160 | 56.248 | 5-23 |  | 965692 | 169.121 | 5-23 | 784726 | 179.887 | 5-23 |
| Mumps | 406961 | 37.779 | 5-23 | 248252 | 30.459 | 5-23 |  | 349196 | 59.898 | 4-23 | 216236 | 47.755 | 4-23 |
| Hepatitis B | 160824 | 14.937 | 5-23 | 140241 | 17.183 | 5-23 |  | 87037 | 14.921 | 5-23 | 66670 | 14.701 | 5-23 |
| Rubella | 16916 | 1.581 | 5-23 | 13103 | 1.629 | 5-23 |  | 13503 | 2.332 | 5-23 | 8563 | 1.909 | 5-23 |
| Hepatitis A | 6723 | 0.621 | 5-23 | 4417 | 0.536 | 5-23 |  | 2944 | 0.503 | 5-23 | 2162 | 0.473 | 5-23 |
| Measles | 4156 | 0.384 | 5-23 | 3683 | 0.446 | 5-23 |  | 3399 | 0.58 | 5-23 | 2758 | 0.601 | 5-23 |
| Pertussis | 2036 | 0.194 | 5-23 | 1980 | 0.254 | 5-22 |  | 1941 | 0.341 | 6-22 | 1975 | 0.456 | 6-23 |
| Hepatitis D | 24 | 0.003 | 9-23 | 18 | 0.003 | 8-23 |  | 14 | 0.004 | 7-23 | 6 | 0.003 | 18-23 |
| Diphtheria | - | - | - | - | - | - |  | - | - | - | - | - | - |
| NT | - | - | - | - | - | - |  | - | - | - | - | - | - |
| Poliomyelitis | - | - | - | - | - | - |  | - | - | - | - | - | - |
| **Bacteria** | **376753** | **35.083** |  | **240041** | **29.649** |  |  | **270126** | **46.426** |  | **171694** | **38.069** |  |
| TB | 321194 | 29.91 | 5-24 | 200388 | 24.768 | 5-23 |  | 179790 | 30.9 | 5-23 | 109184 | 24.247 | 5-23 |
| SF | 55161 | 5.136 | 5-23 | 39463 | 4.858 | 5-23 |  | 90162 | 15.496 | 5-23 | 62455 | 13.81 | 5-23 |
| MM | 248 | 0.023 | 6-21 | 97 | 0.012 | 6-22 |  | 109 | 0.019 | 6-23 | 32 | 0.007 | 6-23 |
| Leprosy | 150 | 0.014 | 7-23 | 93 | 0.011 | 7-23 |  | 65 | 0.011 | 7-23 | 23 | 0.005 | 10-23 |
| **Gastrointestil and enterovirus** | **593056** | **55.463** |  | **408962** | **50.823** |  |  | **692587** | **119.705** |  | **482612** | **108.001** |  |
| HFMD | 261673 | 24.411 | 5-23 | 179726 | 22.228 | 5-23 |  | 358606 | 61.821 | 4-23 | 245414 | 54.663 | 5-23 |
| ID | 266870 | 25.06 | 5-23 | 184580 | 23.117 | 5-23 |  | 278502 | 48.365 | 5-23 | 198257 | 44.747 | 5-23 |
| Dysentery | 37419 | 3.471 | 5-23 | 25424 | 3.112 | 5-23 |  | 39072 | 6.701 | 5-23 | 26326 | 5.802 | 5-23 |
| AHC | 21364 | 1.989 | 5-23 | 14345 | 1.767 | 5-23 |  | 12434 | 2.137 | 5-23 | 8808 | 1.948 | 5-23 |
| T/P | 5730 | 0.532 | 5-23 | 4887 | 0.599 | 5-23 |  | 3973 | 0.681 | 5-23 | 3807 | 0.841 | 5-23 |
| **Sexually transmitted and bloodborne** | **160842** | **15.103** |  | **142667** | **17.752** |  |  | **211780** | **36.677** |  | **129665** | **29.053** |  |
| Syphilis | 51429 | 4.841 | 5-23 | 112091 | 13.97 | 6-23 |  | 56016 | 9.729 | 5-23 | 100133 | 22.445 | 5-23 |
| Gonorrhea | 70983 | 6.674 | 5-23 | 11999 | 1.503 | 5-23 |  | 102682 | 17.799 | 6-23 | 18343 | 4.145 | 5-23 |
| HIV/AIDS | 30074 | 2.814 | 5-23 | 10406 | 1.279 | 5-23 |  | 45623 | 7.875 | 5-23 | 4668 | 1.033 | 5-23 |
| Hepatitis C | 8356 | 0.774 | 5-23 | 8171 | 1 | 5-23 |  | 7459 | 1.274 | 6-23 | 6521 | 1.43 | 6-23 |
| **Vectorborne** | **3916** | **0.378** |  | **2502** | **0.334** |  |  | **7295** | **1.278** |  | **5270** | **1.318** |  |
| Dengue | 857 | 0.091 | 6-23 | 584 | 0.082 | 6-23 |  | 6143 | 1.055 | 6-23 | 4627 | 1.147 | 6-23 |
| JE | 1324 | 0.122 | 5-23 | 859 | 0.104 | 6-23 |  | 399 | 0.068 | 6-23 | 232 | 0.051 | 6-22 |
| Typhus | 795 | 0.074 | 6-23 | 759 | 0.094 | 5-23 |  | 301 | 0.052 | 6-23 | 235 | 0.052 | 6-23 |
| Malaria | 527 | 0.049 | 6-23 | 71 | 0.009 | 7-23 |  | 249 | 0.043 | 6-23 | 40 | 0.009 | 6-23 |
| SM | 360 | 0.037 | 6-23 | 173 | 0.038 | 6-22 |  | 178 | 0.055 | 6-23 | 111 | 0.054 | 7-22 |
| Kala, azar | 53 | 0.005 | 6-23 | 56 | 0.007 | 6-23 |  | 25 | 0.005 | 6-22 | 25 | 0.005 | 6-22 |
| Filariasis | - | - | - | - | - | - |  | - | - | - | - | - | - |
| **Zoonotic** | **14223** | **1.329** |  | **6516** | **0.809** |  |  | **4927** | **0.852** |  | **2286** | **0.514** |  |
| Brucellosis | 10484 | 0.979 | 5-23 | 3715 | 0.461 | 5-23 |  | 2930 | 0.506 | 5-23 | 1073 | 0.24 | 6-23 |
| Hepatitis E | 1577 | 0.147 | 6-23 | 932 | 0.115 | 6-23 |  | 1464 | 0.252 | 6-23 | 841 | 0.187 | 6-23 |
| HD | 1681 | 0.157 | 6-23 | 1615 | 0.199 | 6-23 |  | 393 | 0.068 | 6-23 | 322 | 0.072 | 6-23 |
| Rabies | 267 | 0.025 | 5-23 | 155 | 0.019 | 6-21 |  | 98 | 0.017 | 6-23 | 31 | 0.008 | 7-22 |
| Anthrax | 121 | 0.011 | 6-23 | 57 | 0.007 | 6-22 |  | 11 | 0.002 | 16-23 | 5 | 0.002 | 7-16 |
| Leptospirosis | 90 | 0.008 | 6-23 | 38 | 0.007 | 6-22 |  | 25 | 0.004 | 6-23 | 9 | 0.003 | 6-22 |
| H7N9 | 3 | 0.002 | 9-21 | 4 | 0.001 | 8-19 |  | 6 | 0.003 | 6-21 | 5 | 0.002 | 7-23 |
| H5N1 | - | - | - | - | - | - |  | - | - | - | - | - | - |
| SARS | - | - | - | - | - | - |  | - | - | - | - | - | - |
| **Quarantinable** | **2883** | **0.269** |  | **1012** | **0.126** |  |  | **1496** | **0.259** |  | **483** | **0.11** |  |
| HF | 2879 | 0.268 | 5-23 | 1011 | 0.125 | 5-23 |  | 1491 | 0.257 | 6-23 | 479 | 0.108 | 6-23 |
| Cholera | 4 | 0.001 | 10-22 | 1 | 0.001 | 21-21 |  | 5 | 0.002 | 19-23 | 4 | 0.002 | 19-22 |
| Plague | - | - | - | - | - | - |  | - | - | - | - | - | - |

**Note:** *Total numbers from 2013 to 2021; # Average incidence 2013 to 2021; -, no cases. SI, Seasonal influenza; NT, Neonatal tetanus; TB, Tuberculosis; SF, Scarlet fever; MM, Meningococcal meningitis; T/P, typhoid fever and Paratyphoid fever; HFMD, Hand, foot, and mouth disease; AHC, Acute hemorrhagic conjunctivitis; ID, Infectious diarrhea; AIDS, Acquired Immune Deficiency Syndrome; SM, Schistosomiasis; JE, Japanese encephalitis; HD, Hydatid disease; SARS, severe acute respiratory syndrome; HF, Hemorrhagic fever.
