# Supplementary material for: Long-term variations of urban–Rural disparities in infectious disease burden of over 8.44 million children, adolescents, and youth in China from 2013 to 2021: An observational study
Source: PLoS Med. 2024 Apr 12;21(4):e1004374. doi: 10.1371/journal.pmed.1004374 (PMC11014433; doi:10.1371/journal.pmed.1004374)
Supplement: S2 Table — Note: IRR, incidence rate ratio. (DOCX) [file pmed.1004374.s007.docx]

| **S2 Table.** Trends in incidence and disparity of incidence rate ratio (IRR) of notifiable infectious diseases between urban and rural children, adolescents, and youth from 2013 to 2021. | | | | | | | | | | |
| --- | --- | --- | --- | --- | --- | --- | --- | --- | --- | --- |
| **Year** | All incidence | | | | | All incidence without seasonal influenza | | | | |
|  | **Urban** | **Rural** | **IRR** | **95%CI of IRR** | **p value** | **Urban** | **Rural** | **IRR** | **95%CI of IRR** | **p value** |
| 2013 | 292.757 | 182.892 | 1.601 | 1.593,1.608 | <0.001 | 275.702 | 175.551 | 1.570 | 1.563,1.578 | <0.001 |
| 2014 | 300.851 | 172.294 | 1.746 | 1.738,1.754 | <0.001 | 271.886 | 163.145 | 1.667 | 1.658,1.675 | <0.001 |
| 2015 | 255.276 | 150.983 | 1.691 | 1.682,1.699 | <0.001 | 232.774 | 142.735 | 1.631 | 1.622,1.639 | <0.001 |
| 2016 | 295.544 | 157.43 | 1.877 | 1.868,1.886 | <0.001 | 246.142 | 144.515 | 1.703 | 1.695,1.712 | <0.001 |
| 2017 | 351.879 | 181.504 | 1.939 | 1.93,1.947 | <0.001 | 268.018 | 158.82 | 1.688 | 1.679,1.696 | <0.001 |
| 2018 | 405.686 | 205.185 | 1.977 | 1.969,1.985 | <0.001 | 299.518 | 172.554 | 1.736 | 1.728,1.744 | <0.001 |
| 2019 | 1212.072 | 449.781 | 2.695 | 2.688,2.702 | <0.001 | 344.935 | 199.904 | 1.726 | 1.718,1.733 | <0.001 |
| 2020 | 387.806 | 186.962 | 2.074 | 2.065,2.083 | <0.001 | 184.239 | 118.105 | 1.56 | 1.551,1.569 | <0.001 |
| 2021 | 454.616 | 216.696 | 2.098 | 2.089,2.107 | <0.001 | 269.328 | 141.571 | 1.902 | 1.892,1.912 | <0.001 |

**Note:** IRR, incidence rate ratio.
