# Supplementary material for: Long-term variations of urban–Rural disparities in infectious disease burden of over 8.44 million children, adolescents, and youth in China from 2013 to 2021: An observational study
Source: PLoS Med. 2024 Apr 12;21(4):e1004374. doi: 10.1371/journal.pmed.1004374 (PMC11014433; doi:10.1371/journal.pmed.1004374)
Supplement: S3 Table — Note: IRR, incidence rate ratio. (DOCX) [file pmed.1004374.s008.docx]

| **S3 Table**. Trends in incidence and disparity of incidence rate ratio (IRR) of notifiable infectious diseases by category between urban and rural children, adolescents, and youth from 2013 to 2021. | | | | | |  |
| --- | --- | --- | --- | --- | --- | --- |
| **Category** | **year** | **Urban** | **Rural** | **IRR** | **95%CI of IRR** | **p value** |
| Vaccine preventable | 2013 | 133.418 | 90.637 | 1.472 | 1.462,1.482 | <0.001 |
| Vaccine preventable | 2014 | 101.277 | 62.176 | 1.629 | 1.616,1.642 | <0.001 |
| Vaccine preventable | 2015 | 87.686 | 57.047 | 1.537 | 1.524,1.550 | <0.001 |
| Vaccine preventable | 2016 | 108.602 | 56.754 | 1.914 | 1.899,1.929 | <0.001 |
| Vaccine preventable | 2017 | 163.088 | 81.842 | 1.993 | 1.980,2.006 | <0.001 |
| Vaccine preventable | 2018 | 187.856 | 92.593 | 2.029 | 2.017,2.041 | <0.001 |
| Vaccine preventable | 2019 | 973.000 | 328.577 | 2.961 | 2.952,2.970 | <0.001 |
| Vaccine preventable | 2020 | 241.238 | 97.113 | 2.484 | 2.470,2.499 | <0.001 |
| Vaccine preventable | 2021 | 225.013 | 100.176 | 2.246 | 2.233,2.260 | <0.001 |
| Bacteria | 2013 | 41.330 | 33.232 | 1.244 | 1.230,1.258 | <0.001 |
| Bacteria | 2014 | 48.704 | 36.048 | 1.351 | 1.336,1.366 | <0.001 |
| Bacteria | 2015 | 48.527 | 34.992 | 1.387 | 1.372,1.402 | <0.001 |
| Bacteria | 2016 | 44.745 | 33.465 | 1.337 | 1.322,1.352 | <0.001 |
| Bacteria | 2017 | 47.726 | 35.204 | 1.356 | 1.341,1.371 | <0.001 |
| Bacteria | 2018 | 46.951 | 33.322 | 1.409 | 1.393,1.425 | <0.001 |
| Bacteria | 2019 | 50.379 | 36.839 | 1.368 | 1.353,1.383 | <0.001 |
| Bacteria | 2020 | 28.121 | 26.909 | 1.045 | 1.031,1.060 | <0.001 |
| Bacteria | 2021 | 28.353 | 24.570 | 1.154 | 1.137,1.171 | <0.001 |
| Gastrointestinal and enterovirus | 2013 | 90.885 | 45.559 | 1.995 | 1.978,2.012 | <0.001 |
| Gastrointestinal and enterovirus | 2014 | 114.241 | 58.667 | 1.947 | 1.932,1.962 | <0.001 |
| Gastrointestinal and enterovirus | 2015 | 87.909 | 43.190 | 2.035 | 2.017,2.054 | <0.001 |
| Gastrointestinal and enterovirus | 2016 | 112.143 | 51.686 | 2.170 | 2.152,2.187 | <0.001 |
| Gastrointestinal and enterovirus | 2017 | 107.701 | 47.151 | 2.284 | 2.265,2.303 | <0.001 |
| Gastrointestinal and enterovirus | 2018 | 136.427 | 61.328 | 2.225 | 2.208,2.241 | <0.001 |
| Gastrointestinal and enterovirus | 2019 | 147.960 | 63.322 | 2.337 | 2.320,2.354 | <0.001 |
| Gastrointestinal and enterovirus | 2020 | 81.242 | 42.797 | 1.898 | 1.881,1.916 | <0.001 |
| Gastrointestinal and enterovirus | 2021 | 152.775 | 67.450 | 2.265 | 2.249,2.282 | <0.001 |
| Sexually transmitted and bloodborne | 2013 | 25.399 | 11.601 | 2.189 | 2.153,2.226 | <0.001 |
| Sexually transmitted and bloodborne | 2014 | 29.882 | 13.473 | 2.218 | 2.183,2.253 | <0.001 |
| Sexually transmitted and bloodborne | 2015 | 29.416 | 13.685 | 2.150 | 2.116,2.183 | <0.001 |
| Sexually transmitted and bloodborne | 2016 | 28.788 | 13.922 | 2.068 | 2.036,2.100 | <0.001 |
| Sexually transmitted and bloodborne | 2017 | 31.920 | 15.800 | 2.020 | 1.991,2.050 | <0.001 |
| Sexually transmitted and bloodborne | 2018 | 33.064 | 16.635 | 1.988 | 1.959,2.017 | <0.001 |
| Sexually transmitted and bloodborne | 2019 | 38.224 | 19.345 | 1.976 | 1.949,2.003 | <0.001 |
| Sexually transmitted and bloodborne | 2020 | 36.275 | 18.800 | 1.930 | 1.903,1.957 | <0.001 |
| Sexually transmitted and bloodborne | 2021 | 47.266 | 22.970 | 2.058 | 2.032,2.084 | <0.001 |
| Vectorborne | 2013 | 0.863 | 0.523 | 1.649 | 1.516,1.794 | <0.001 |
| Vectorborne | 2014 | 5.785 | 0.389 | 14.863 | 13.824,15.980 | <0.001 |
| Vectorborne | 2015 | 0.736 | 0.464 | 1.586 | 1.447,1.739 | <0.001 |
| Vectorborne | 2016 | 0.352 | 0.272 | 1.296 | 1.142,1.472 | <0.001 |
| Vectorborne | 2017 | 0.590 | 0.297 | 1.988 | 1.784,2.215 | <0.001 |
| Vectorborne | 2018 | 0.592 | 0.255 | 2.323 | 2.076,2.599 | <0.001 |
| Vectorborne | 2019 | 1.758 | 0.554 | 3.176 | 2.952,3.418 | <0.001 |
| Vectorborne | 2020 | 0.134 | 0.160 | 0.832 | 0.686,1.010 | 0.062 |
| Vectorborne | 2021 | 0.096 | 0.140 | 0.687 | 0.548,0.863 | 0.001 |
| Zoonotic | 2013 | 0.653 | 1.079 | 0.605 | 0.558,0.656 | <0.001 |
| Zoonotic | 2014 | 0.775 | 1.324 | 0.585 | 0.543,0.631 | <0.001 |
| Zoonotic | 2015 | 0.807 | 1.395 | 0.578 | 0.537,0.622 | <0.001 |
| Zoonotic | 2016 | 0.782 | 1.178 | 0.663 | 0.615,0.716 | <0.001 |
| Zoonotic | 2017 | 0.661 | 0.995 | 0.664 | 0.612,0.721 | <0.001 |
| Zoonotic | 2018 | 0.591 | 0.810 | 0.729 | 0.668,0.797 | <0.001 |
| Zoonotic | 2019 | 0.597 | 0.972 | 0.614 | 0.563,0.671 | <0.001 |
| Zoonotic | 2020 | 0.636 | 1.001 | 0.636 | 0.584,0.692 | <0.001 |
| Zoonotic | 2021 | 0.812 | 1.179 | 0.689 | 0.637,0.745 | <0.001 |
| Quarantinable | 2013 | 0.209 | 0.260 | 0.803 | 0.692,0.932 | 0.004 |
| Quarantinable | 2014 | 0.187 | 0.216 | 0.865 | 0.736,1.017 | 0.079 |
| Quarantinable | 2015 | 0.196 | 0.210 | 0.933 | 0.796,1.095 | 0.394 |
| Quarantinable | 2016 | 0.133 | 0.153 | 0.865 | 0.714,1.048 | 0.139 |
| Quarantinable | 2017 | 0.193 | 0.215 | 0.895 | 0.763,1.050 | 0.173 |
| Quarantinable | 2018 | 0.205 | 0.242 | 0.846 | 0.725,0.986 | 0.033 |
| Quarantinable | 2019 | 0.154 | 0.171 | 0.900 | 0.750,1.080 | 0.257 |
| Quarantinable | 2020 | 0.160 | 0.182 | 0.876 | 0.733,1.048 | 0.147 |
| Quarantinable | 2021 | 0.301 | 0.212 | 1.422 | 1.229,1.646 | <0.001 |

**Note:** IRR, incidence rate ratio.
