# Supplementary material for: Long-term variations of urban–Rural disparities in infectious disease burden of over 8.44 million children, adolescents, and youth in China from 2013 to 2021: An observational study
Source: PLoS Med. 2024 Apr 12;21(4):e1004374. doi: 10.1371/journal.pmed.1004374 (PMC11014433; doi:10.1371/journal.pmed.1004374)
Supplement: S4 Table — Note: IRR, incidence rate ratio. (DOCX) [file pmed.1004374.s009.docx]

**S4 Table.** The incidence and disparity of incidence rate ratio (IRR) of notifiable infectious diseases by category between urban and rural children, adolescents, and youth in eastern, middle, northeastern and western regions from 2013 to 2021.

| Classification | Region | Rural | Urban | IRR | **95%CI of IRR** | **p value** |
| --- | --- | --- | --- | --- | --- | --- |
| Overall | East | 281.57 | 467.27 | 1.660 | 1.656,1.663 | <0.001 |
|  | Middle | 237.73 | 405.01 | 1.704 | 1.699,1.708 | <0.001 |
|  | Northeast | 133.99 | 229.48 | 1.713 | 1.700,1.726 | <0.001 |
|  | West | 256.90 | 333.00 | 1.296 | 1.293,1.300 | <0.001 |
| Vaccine preventable | East | 135.86 | 254.75 | 1.877 | 1.871,1.882 | <0.001 |
|  | Middle | 144.99 | 266.96 | 1.843 | 1.836,1.849 | <0.001 |
|  | Northeast | 40.18 | 66.58 | 1.658 | 1.633,1.684 | <0.001 |
|  | West | 111.20 | 167.22 | 1.506 | 1.501,1.512 | <0.001 |
| Bacteria | East | 26.77 | 36.78 | 1.374 | 1.365,1.383 | <0.001 |
|  | Middle | 26.24 | 31.79 | 1.212 | 1.201,1.222 | <0.001 |
|  | Northeast | 41.85 | 70.00 | 1.673 | 1.651,1.695 | <0.001 |
|  | West | 56.72 | 51.90 | 0.915 | 0.909,0.921 | <0.001 |
| Gastrointestinal and enterovirus | East | 98.49 | 139.98 | 1.421 | 1.416,1.427 | <0.001 |
|  | Middle | 54.82 | 85.87 | 1.566 | 1.557,1.576 | <0.001 |
|  | Northeast | 37.13 | 60.66 | 1.634 | 1.608,1.660 | <0.001 |
|  | West | 63.07 | 81.74 | 1.296 | 1.289,1.303 | <0.001 |
| Sexually transmitted and bloodborne | East | 20.36 | 34.21 | 1.681 | 1.668,1.694 | <0.001 |
|  | Middle | 11.19 | 19.53 | 1.746 | 1.725,1.766 | <0.001 |
|  | Northeast | 20.74 | 36.54 | 1.762 | 1.729,1.797 | <0.001 |
|  | West | 25.06 | 31.05 | 1.239 | 1.229,1.250 | <0.001 |
| Vectorborne | East | 1.21 | 2.55 | 2.105 | 1.988,2.228 | <0.001 |
|  | Middle | 2.99 | 3.33 | 1.116 | 1.011,1.232 | 0.030 |
|  | Northeast | 2.44 | 2.45 | 1.004 | 0.636,1.584 | 0.986 |
|  | West | 3.07 | 4.00 | 1.302 | 1.240,1.368 | <0.001 |
| Zoonotic | East | 1.38 | 1.36 | 0.987 | 0.929,1.050 | 0.675 |
|  | Middle | 1.47 | 1.59 | 1.081 | 1.008,1.159 | 0.029 |
|  | Northeast | 4.60 | 4.56 | 0.990 | 0.906,1.083 | 0.825 |
|  | West | 7.20 | 3.48 | 0.484 | 0.466,0.502 | <0.001 |
| Quarantinable | East | 1.11 | 0.97 | 0.878 | 0.785,0.981 | 0.022 |
|  | Middle | 0.99 | 0.90 | 0.907 | 0.781,1.053 | 0.200 |
|  | Northeast | 3.02 | 3.96 | 1.312 | 1.163,1.480 | <0.001 |
|  | West | 2.35 | 2.12 | 0.903 | 0.829,0.983 | 0.019 |

**Note:** IRR, incidence rate ratio.
