# Supplementary figures and images for: Long-term variations of urban–Rural disparities in infectious disease burden of over 8.44 million children, adolescents, and youth in China from 2013 to 2021: An observational study
Source: PLoS Med. 2024 Apr 12;21(4):e1004374. doi: 10.1371/journal.pmed.1004374 (PMC11014433; doi:10.1371/journal.pmed.1004374)

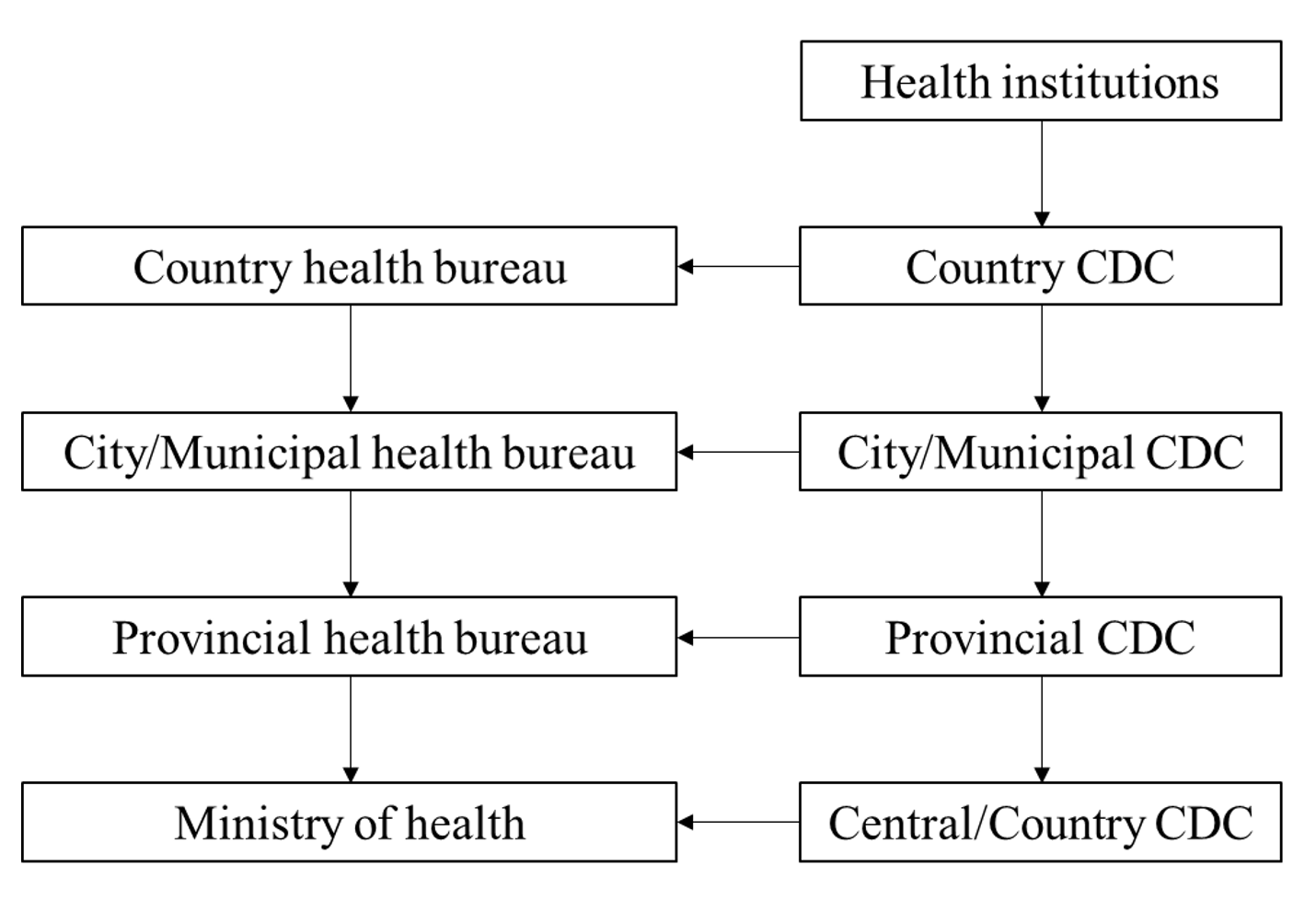

Supplement: S1 Fig — Notes: CDC, Chinese Centers for Disease Control. (TIF) [file pmed.1004374.s010.tif]

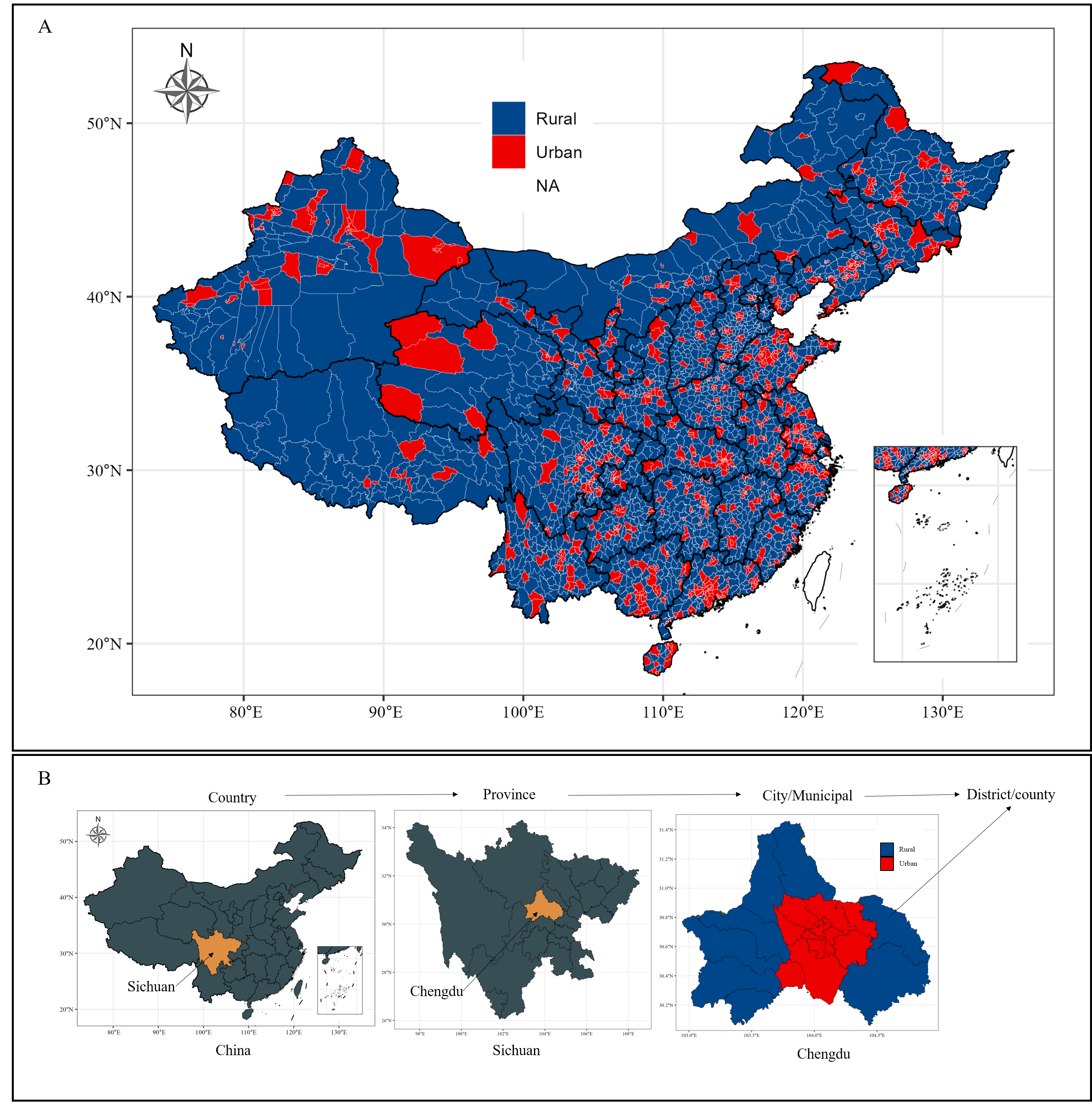

Supplement: S2 Fig — Notes: Subfigure A presents the distribution of urban and rural areas in China; subfigure B presents the schematic diagram of the 3 levels of administrative division in China, which is based on the Sichuan province and Chengdu city. The base map was obtained from Natural Earth (https://naturalearthdata.com). (TIF) [file pmed.1004374.s011.tif]

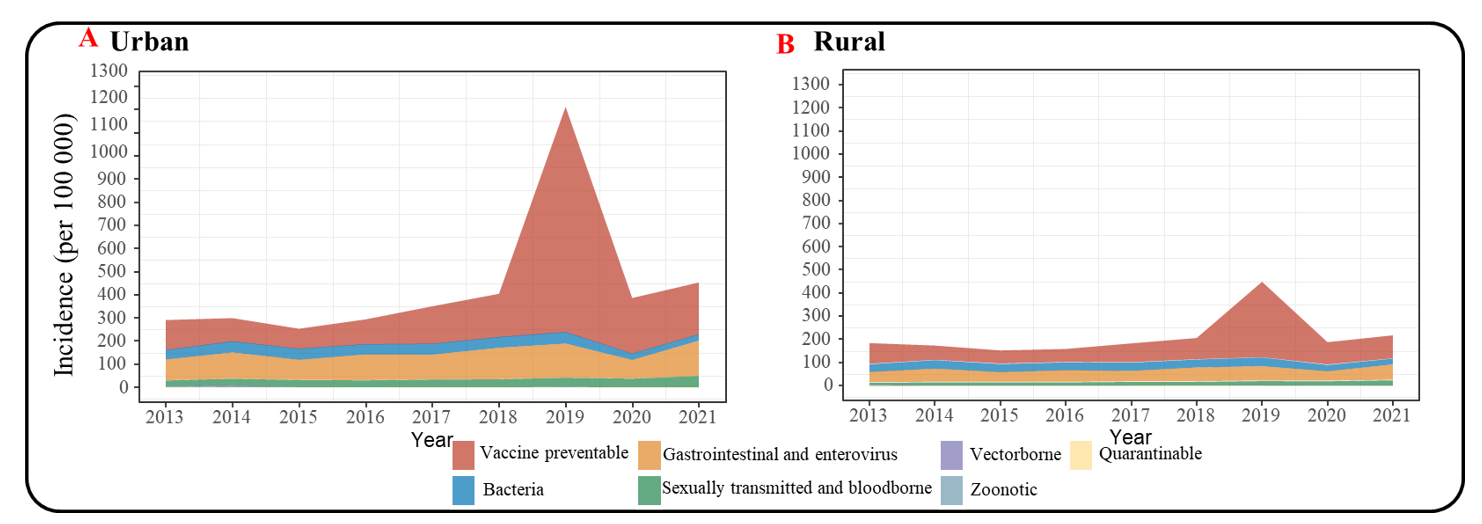

Supplement: S3 Fig — (TIF) [file pmed.1004374.s012.tif]

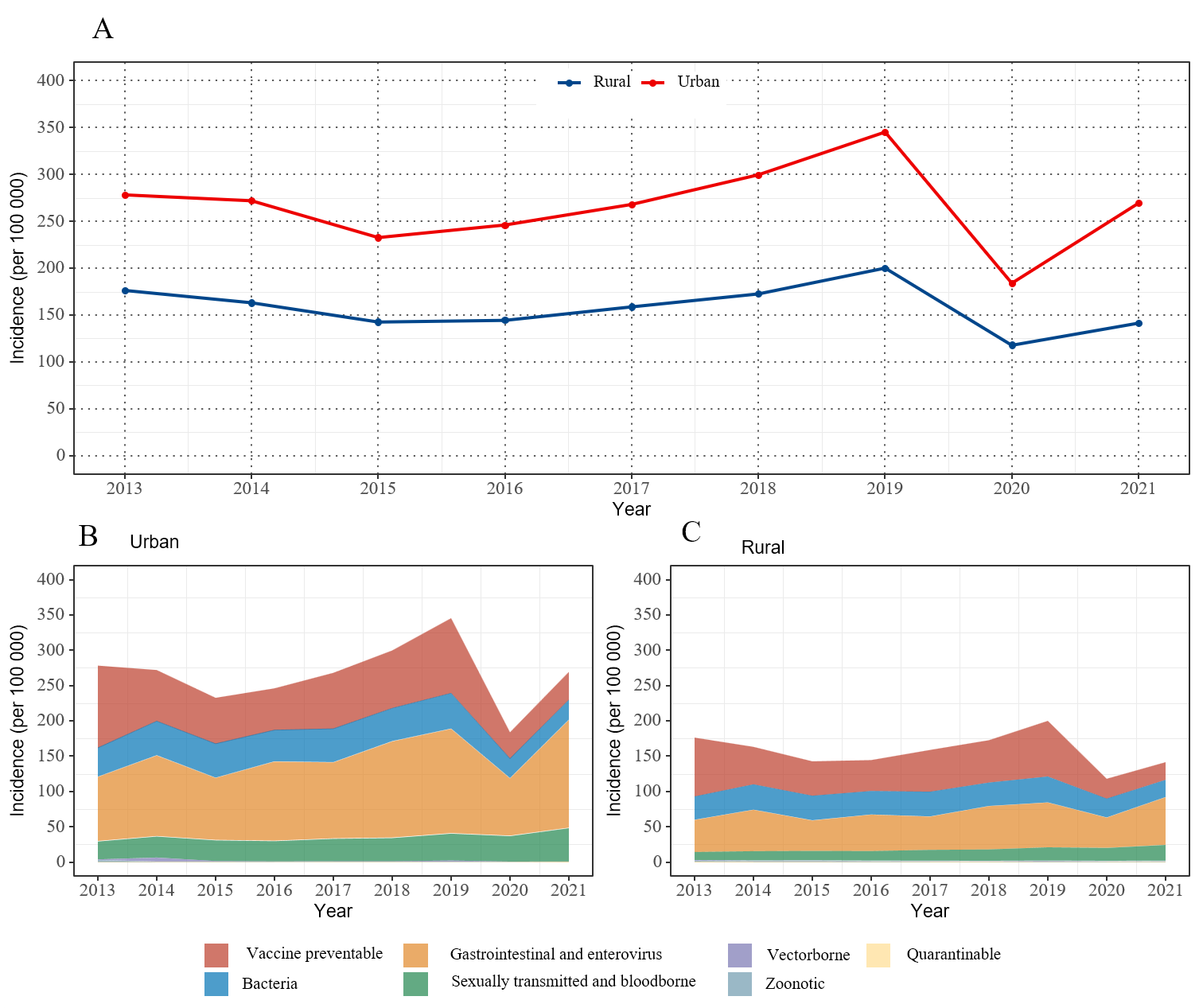

Supplement: S4 Fig — Notes: Incidence calculations for specific infectious disease categories are based on excluding cases of seasonal influenza; subfigure A presents the incidence of overall infectious diseases excluding the seasonal influenza by urban and rural areas; subfigure B presents the total incidence by 7 categories from 2013 to 2021 in urban areas, while subfigure C presents the total incidence by 7 categories over the same period in rural areas. (TIF) [file pmed.1004374.s013.tif]

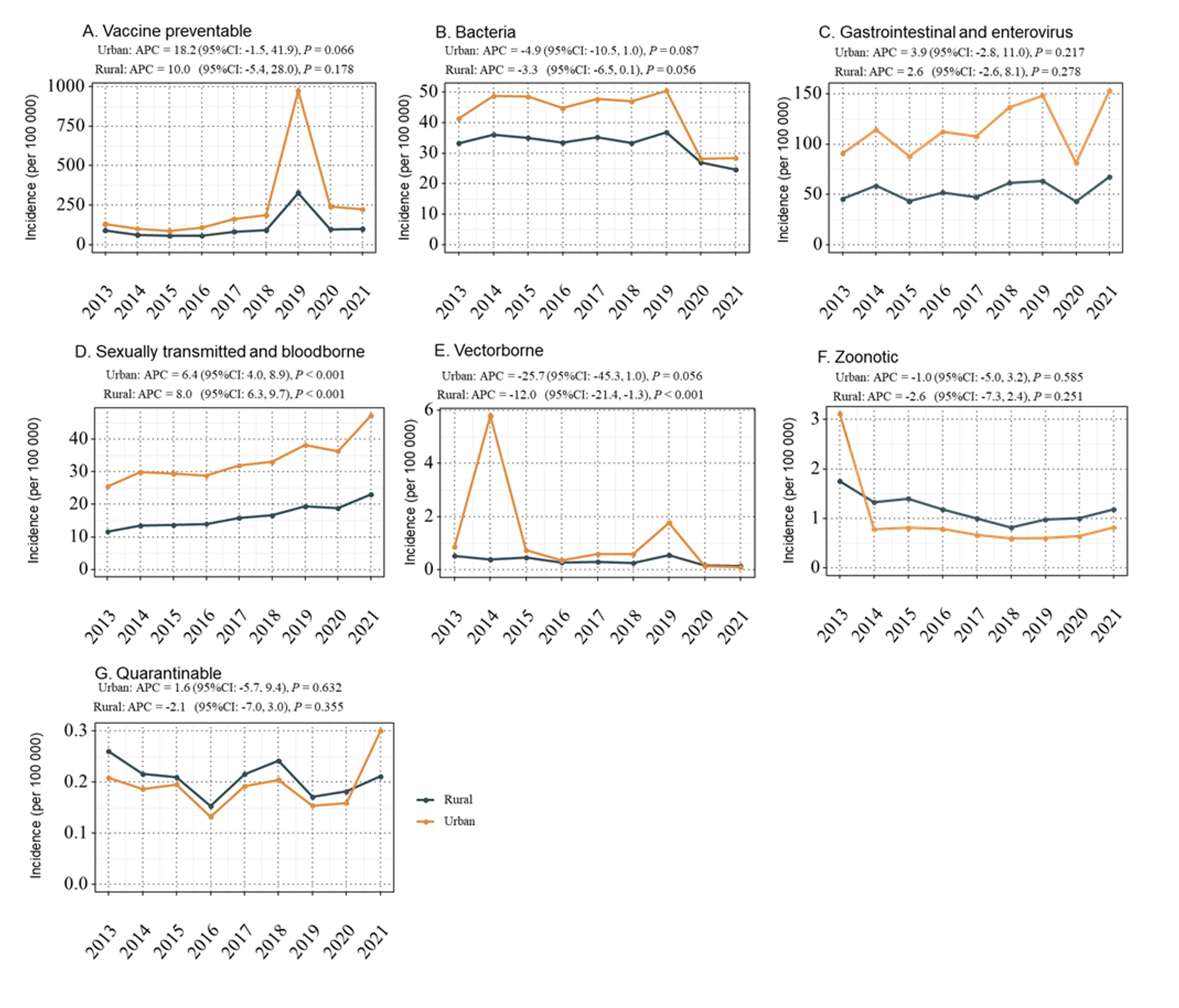

Supplement: S5 Fig — Notes: subfigure A–G presents the incidence of vaccine preventable, bacteria, gastrointestil and enterovirus, sexually transmitted and bloodborne, vectorborne, zoonotic, and quarantinable infectious diseases, respectively. The APC for the incidence in urban and rural areas is shown at the top of each subfigure. (TIF) [file pmed.1004374.s014.tif]

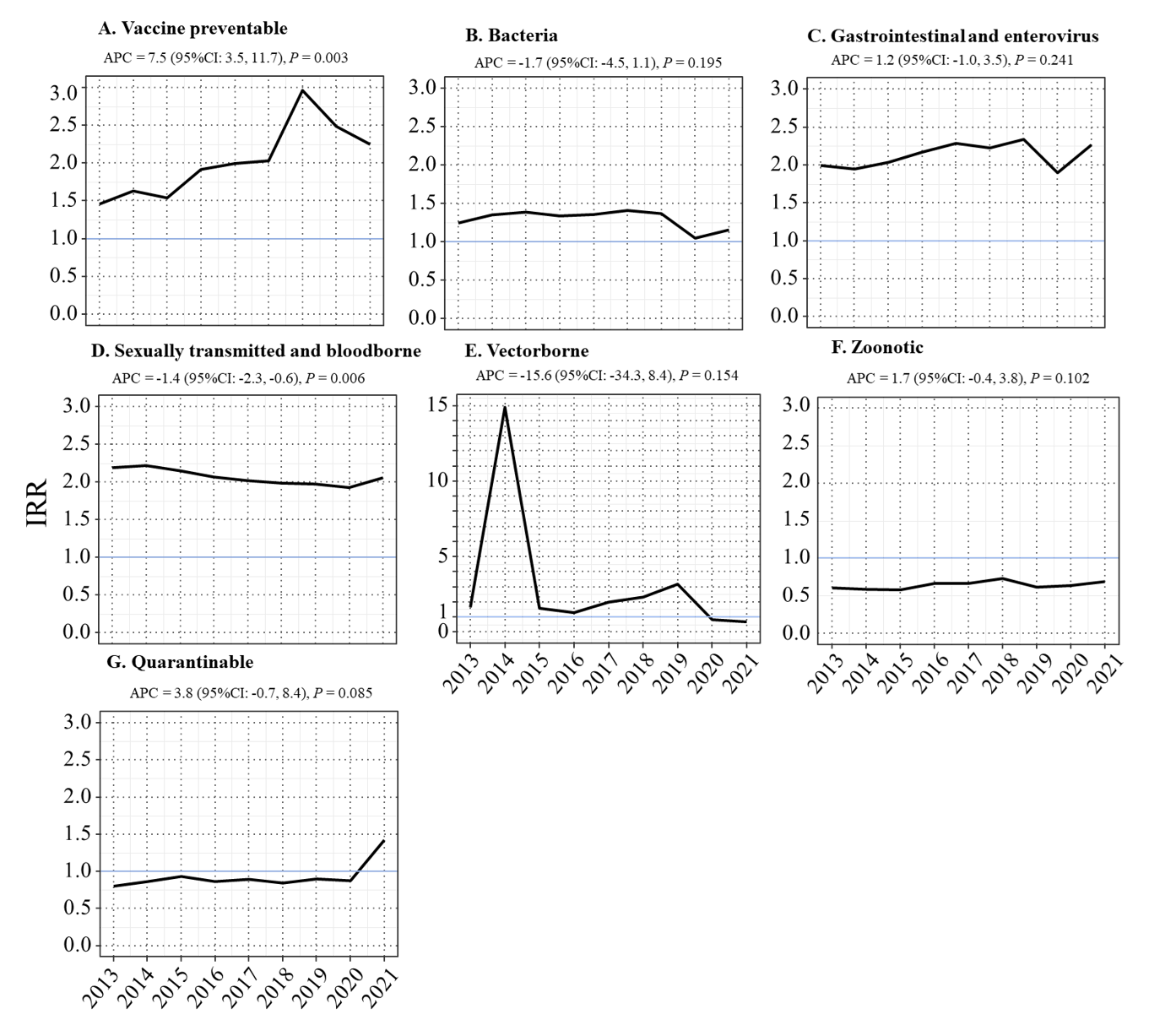

Supplement: S6 Fig — Notes: Subfigure A–G presents the IRR of vaccine preventable, bacteria, gastrointestil and enterovirus, sexually transmitted and bloodborne, vectorborne, zoonotic, and quarantinable infectious diseases, respectively. The APC for the IRR is shown at the top of each subfigure. (TIF) [file pmed.1004374.s015.tif]

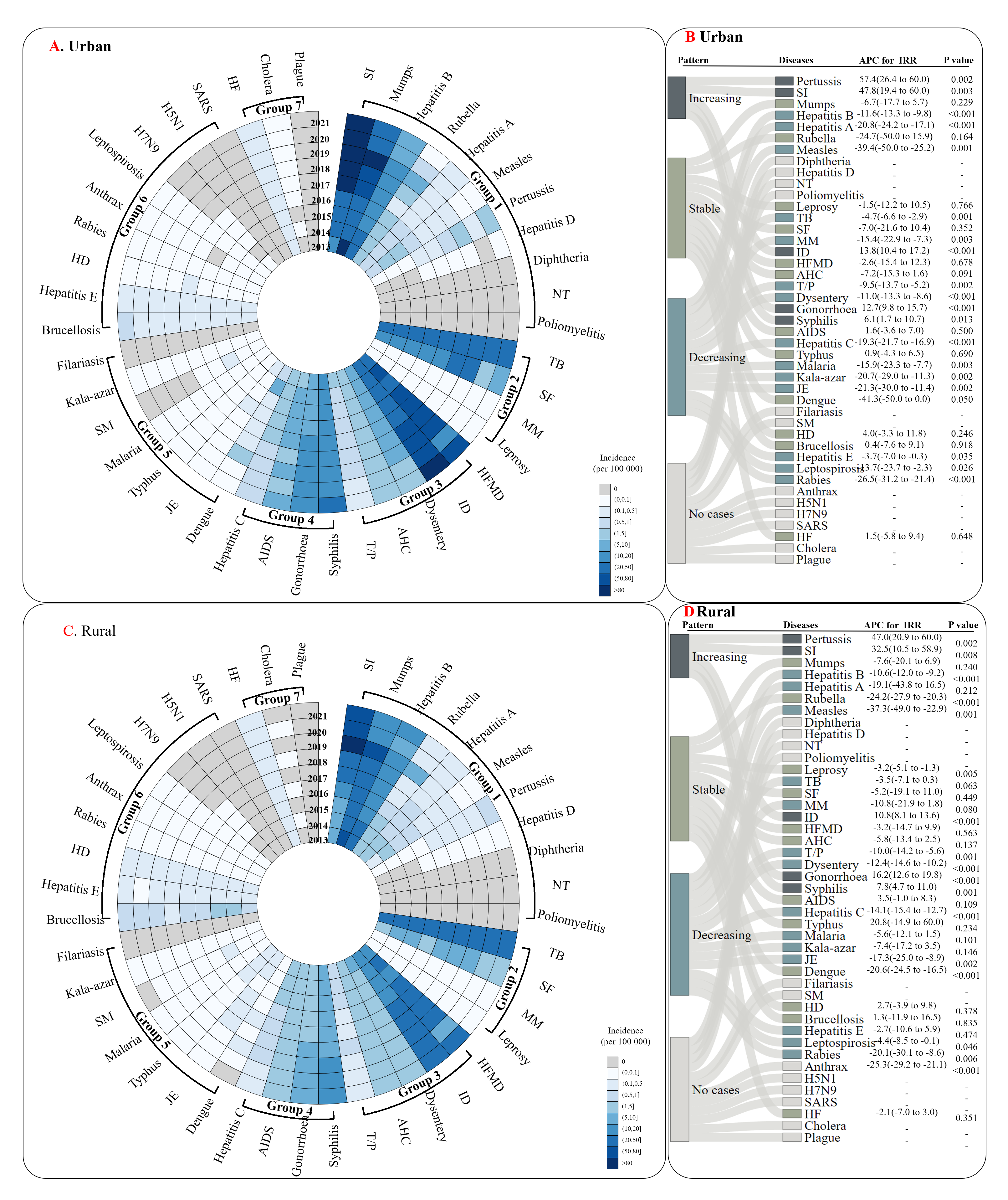

Supplement: S7 Fig — Note: Overall incidence changes for each specific infectious disease between urban and rural (subfigure A for urban and C for rural), and their change patterns based on the annual percentage change (APC) (subfigures B and D). The uppercase letters A–G in the subfigure A represent the 7 categories of infectious disease: Group 1, vaccine preventable; Group 2, bacteria; Group 3, gastrointestil and enterovirus; Group 4, sexually transmitted and bloodborne; Group 5, vectorborne; Group 6, zoonotic; Group 7, quarantinable. The forest plot in the subfigure B represent the APC for IRR, and the actual number of APC (95% CI) and the p value are also represented in the subfigure B. SI, seasonal influenza; NT, neonatal tetanus; TB, tuberculosis; SF, scarlet fever; MM, meningococcal meningitis; T/P, typhoid fever and paratyphoid fever; HFMD, hand, foot, and mouth disease; AHC, acute hemorrhagic conjunctivitis; ID, infectious diarrhea; AIDS, acquired immune deficiency syndrome; SM, schistosomiasis; JE, Japanese encephalitis; HD, hydatid disease; SARS, severe acute respiratory syndrome; HF, hemorrhagic fever; IRR, incidence rate ratio. (TIF) [file pmed.1004374.s016.tif]

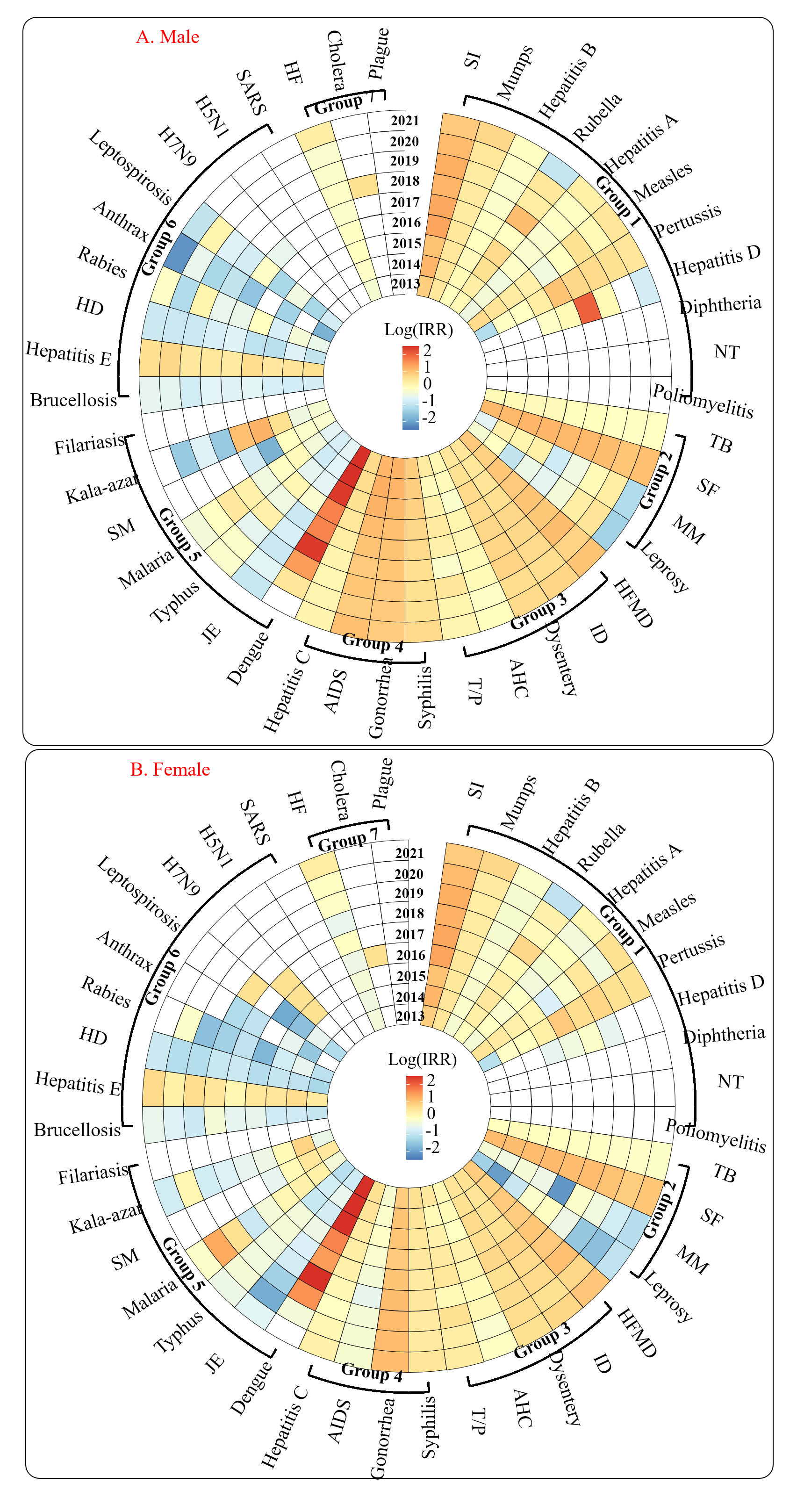

Supplement: S8 Fig — Note: Group 1, vaccine preventable; Group 2, bacteria; Group 3, gastrointestil and enterovirus; Group 4, sexually transmitted and bloodborne; Group 5, vectorborne; Group 6, zoonotic; Group 7, quarantinable. The forest plot in the subfigure B represent the APC for IRR, and the actual number of APC (95% CI) and the p value are also represented in the subfigure B. SI, seasonal influenza; NT, neonatal tetanus; TB, tuberculosis; SF, scarlet fever; MM, meningococcal meningitis; T/P, typhoid fever and paratyphoid fever; HFMD, hand, foot, and mouth disease; AHC, acute hemorrhagic conjunctivitis; ID, infectious diarrhea; AIDS, acquired immune deficiency syndrome; SM, schistosomiasis; JE, Japanese encephalitis; HD, hydatid disease; SARS, severe acute respiratory syndrome; HF, hemorrhagic fever; IRR, incidence rate ratio. (TIF) [file pmed.1004374.s017.tif]

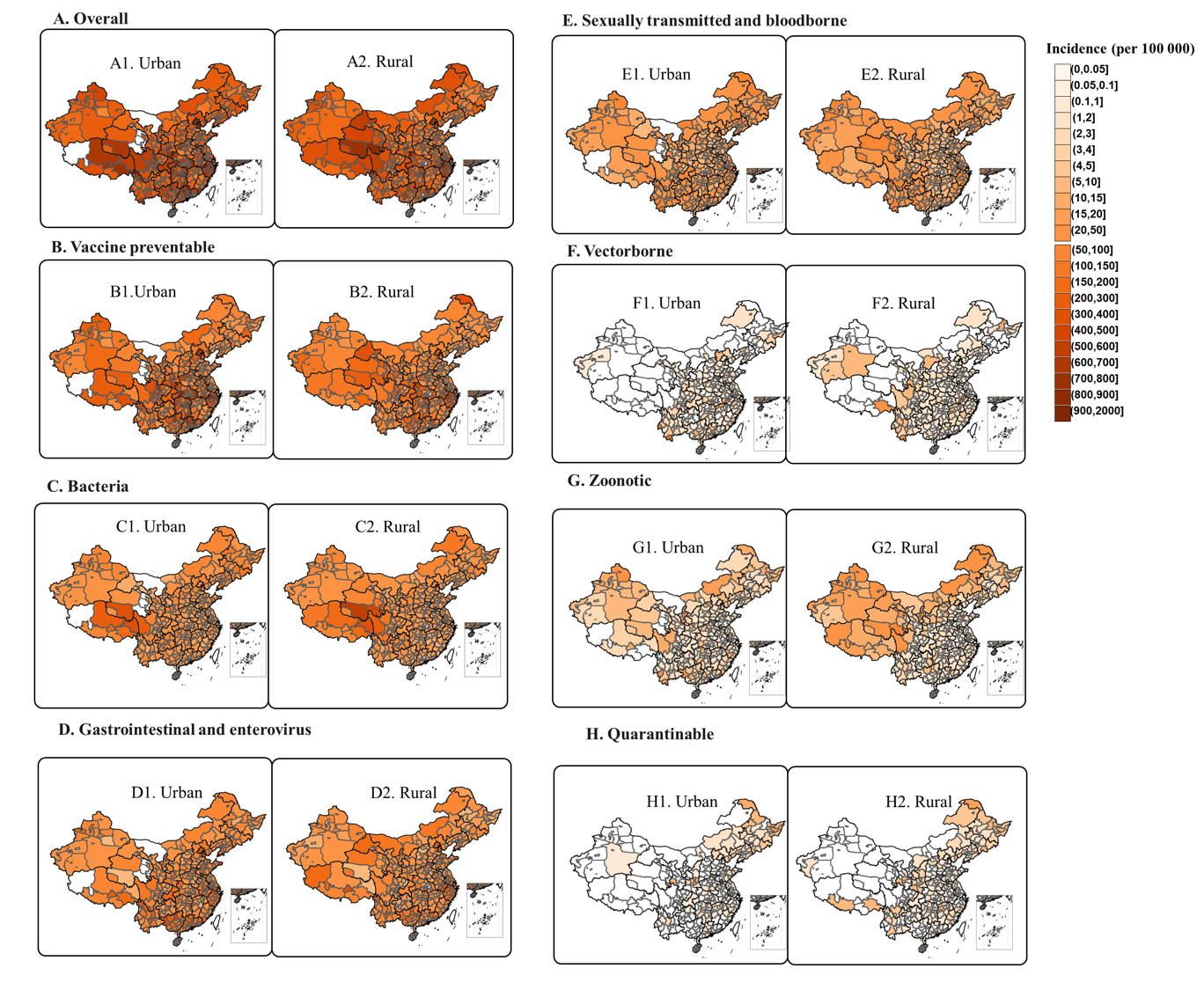

Supplement: S9 Fig — Notes: The uppercase letters A–G in the each panel represent the 7 categories of infectious disease: A, overall infectious disease; B, vaccine preventable; C, bacteria; DD, gastrointestil and enterovirus; E, sexually transmitted and bloodborne; F, vectorborne; G, zoonotic; H, quarantinable. Each panel included 2 figures, the map of incidence in urban and rural; regional distribution of total and specific infectious disease category in incidence was showed for those in urban (subfigure A1–H1) and rural (subfigure A2–H2). The base map was obtained from Natural Earth (https://naturalearthdata.com). (TIF) [file pmed.1004374.s018.tif]

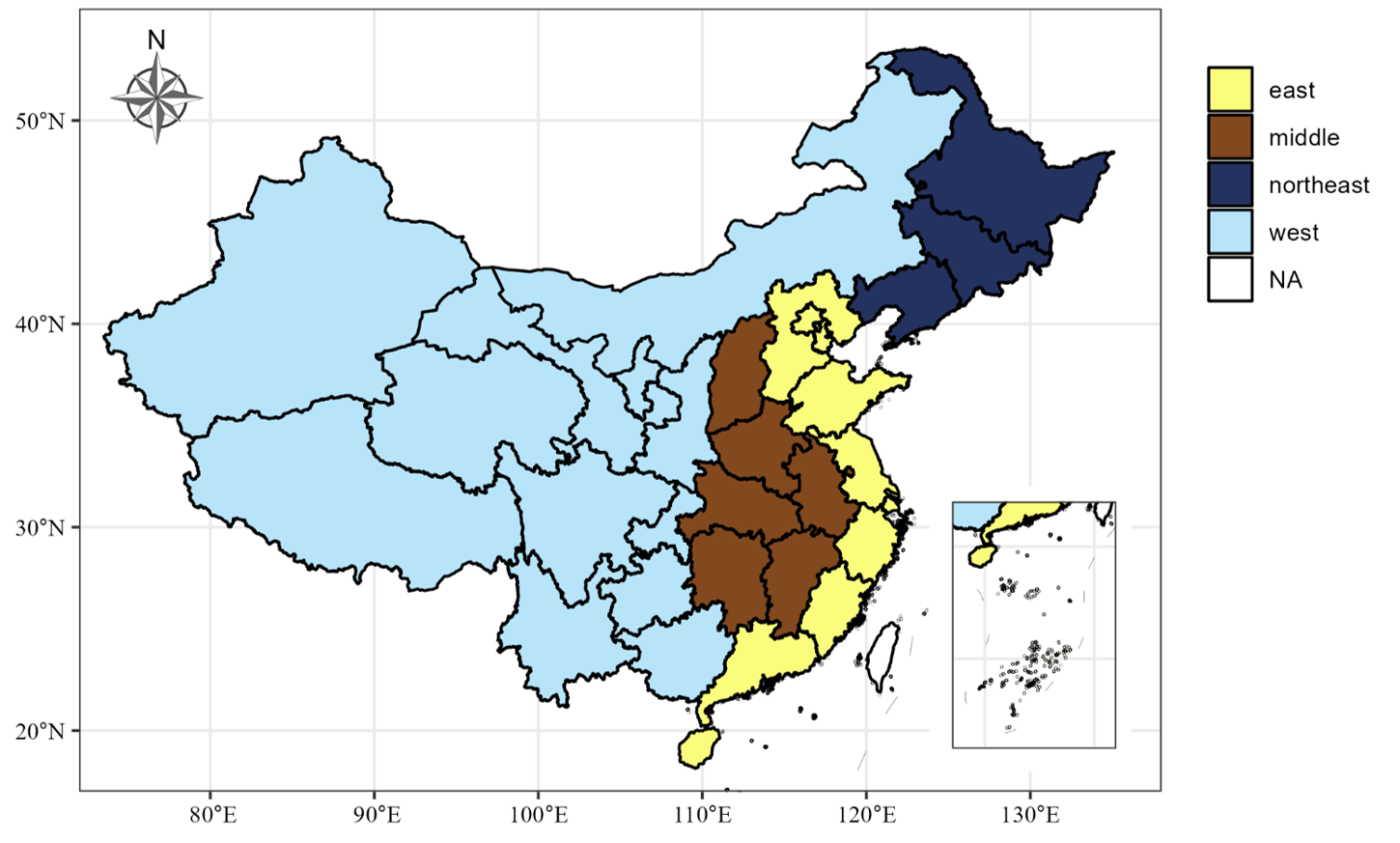

Supplement: S10 Fig — Notes: The base map was obtained from Natural Earth (https://naturalearthdata.com). (TIF) [file pmed.1004374.s019.tif]

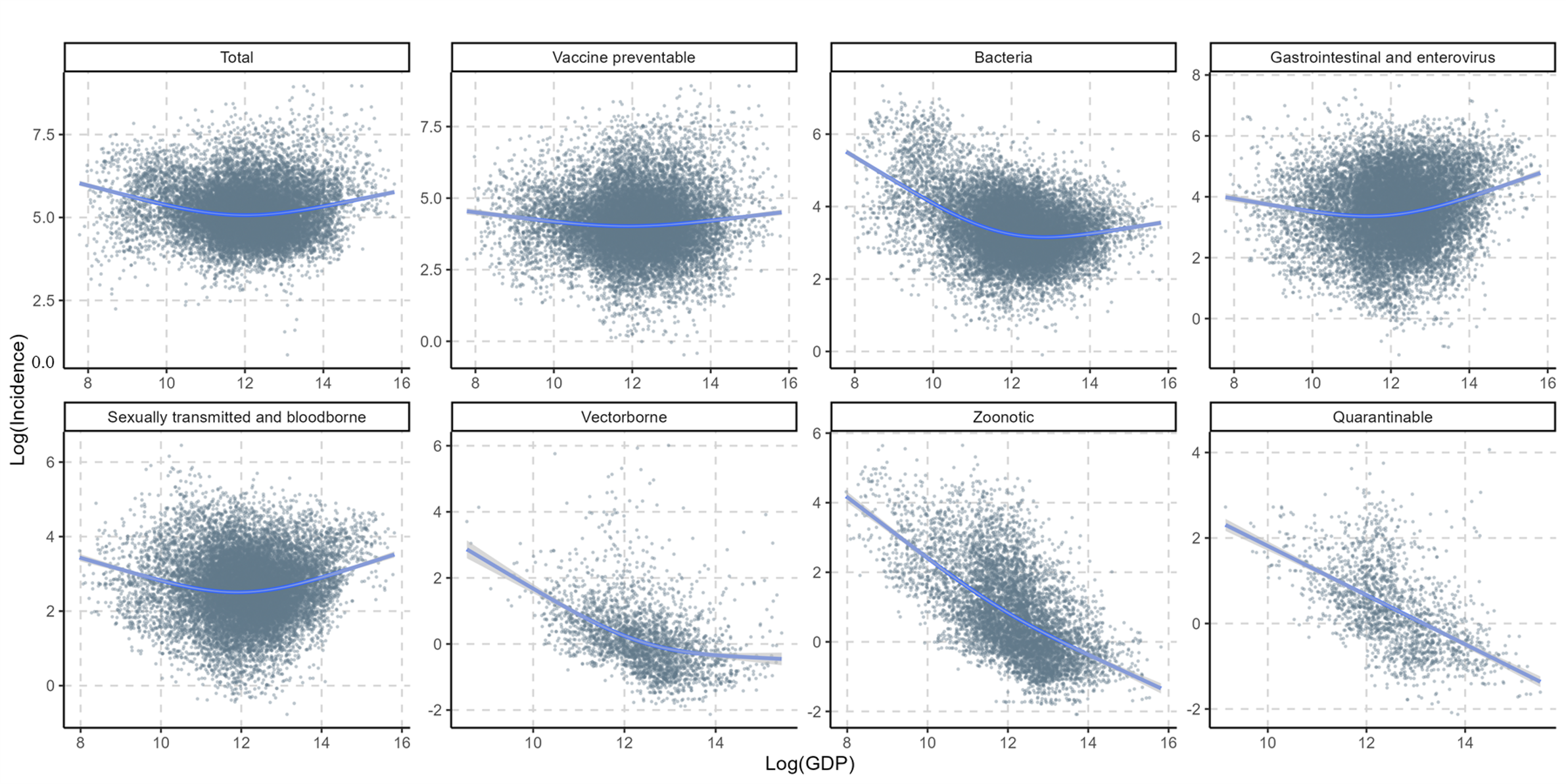

Supplement: S11 Fig — Notes: The log-transform was applied to the incidence and GDP. The blue line using the generalized additive models (GAMs) represents the nonlinear association of GDP and incidence of total and specific categorized notifiable infectious diseases. The dots present the district/county. (TIF) [file pmed.1004374.s020.tif]

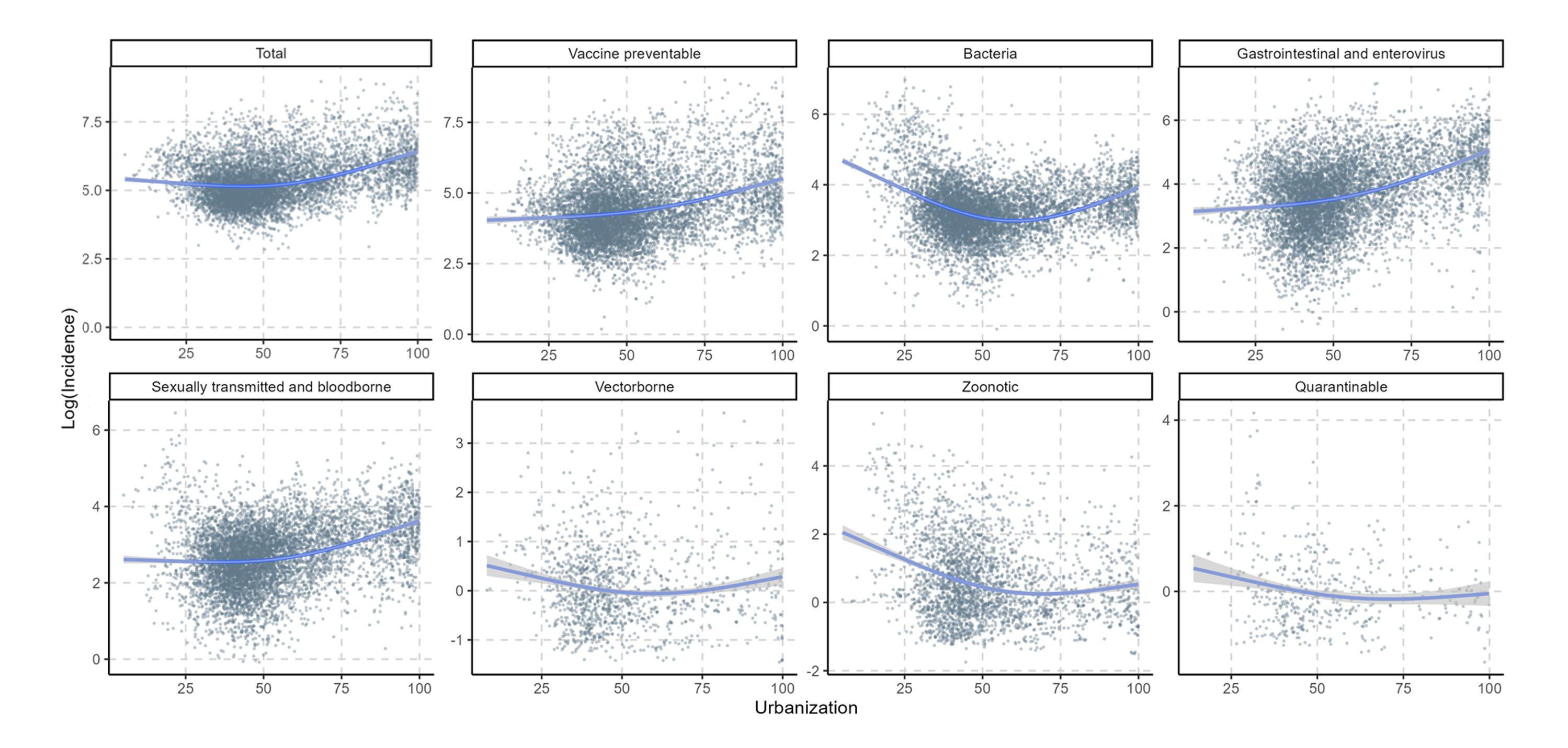

Supplement: S12 Fig — Notes: The log-transform was applied to the incidence and urbanization. The blue line using the generalized additive models (GAMs) represents the nonlinear association of urbanization and incidence of total and specific categorized notifiable infectious diseases. The dots present the district/county. (TIF) [file pmed.1004374.s021.tif]

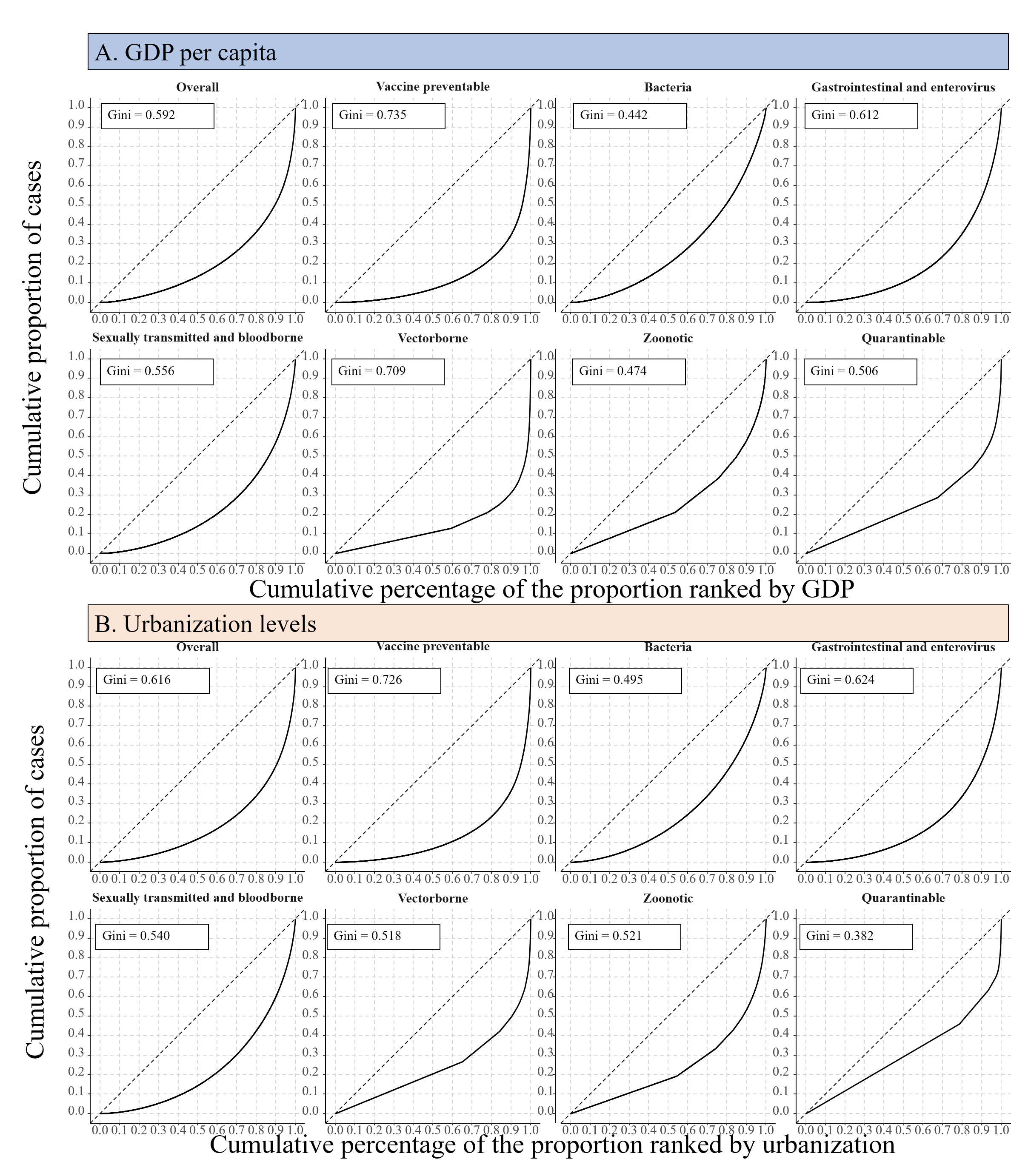

Supplement: S13 Fig — Notes: The (dashed) diagonal line of equality depicted a theoretical scenario where infectious disease cases were equally distributed across the population. The Gini coefficients were calculated based on the Lorenz curves, with larger coefficients indicating more unequal distribution of infectious disease cases and smaller coefficients indicating a more equal distribution. In the subfigure A: The Lorenz curves were generated by sorting the GDP in each city/municipal from the lowest to the highest GDP. The x-axis and y-axis represented the cumulative percentage of the population ranked by the GDP and cumulative proportion of notifiable infectious diseases in the surveillance year. In the subfigure B: The Lorenz curves were generated by sorting the urbanization in each city/municipal from the lowest to the highest urbanization. The x-axis and y-axis represented the cumulative percentage of the population ranked by the urbanization and cumulative proportion of notifiable infectious diseases in the surveillance year. (TIF) [file pmed.1004374.s022.tif]

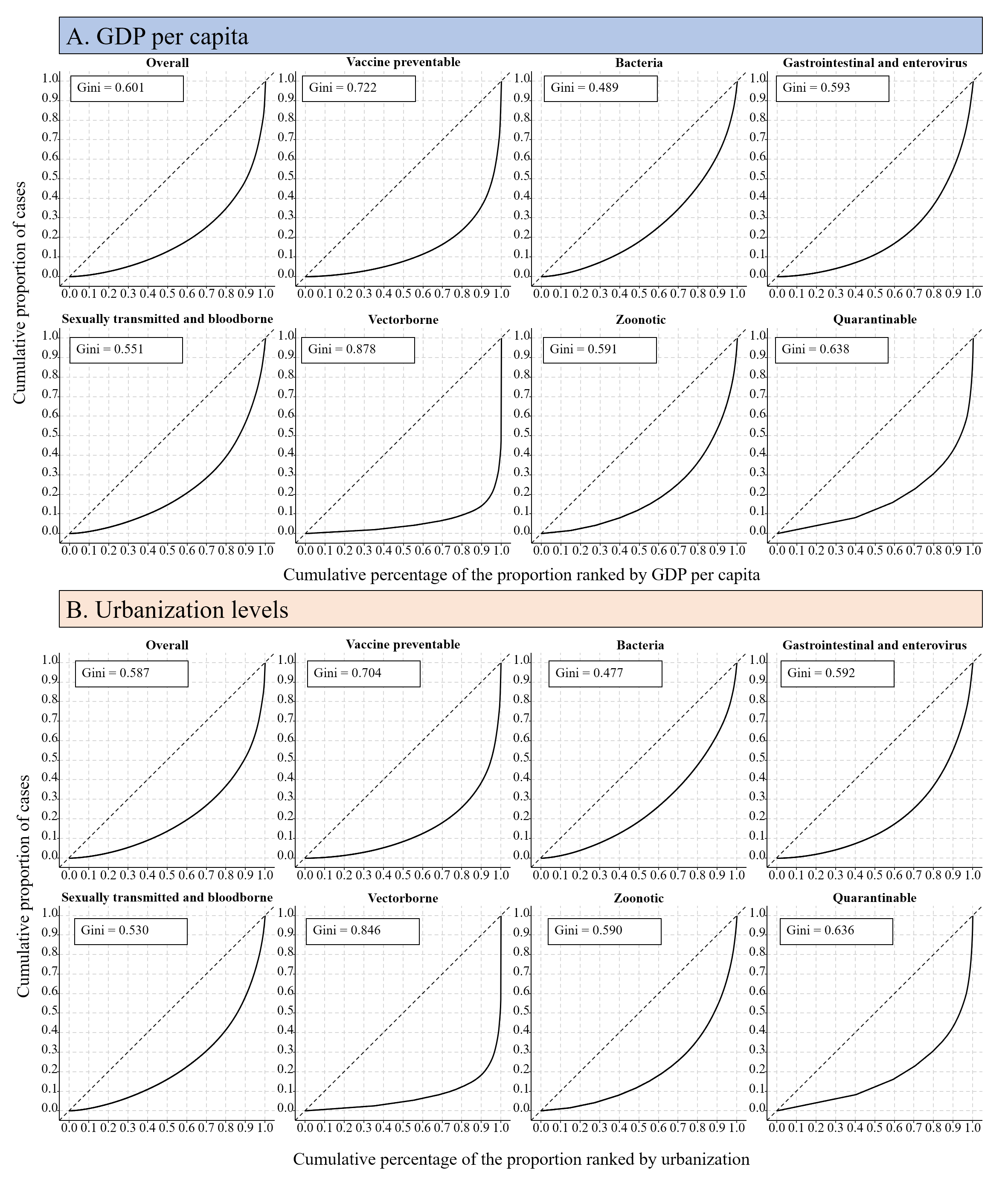

Supplement: S14 Fig — Notes: The (dashed) diagonal line of equality depicted a theoretical scenario where infectious disease cases were equally distributed across the population. The Gini coefficients were calculated based on the Lorenz curves, with larger coefficients indicating more unequal distribution of infectious disease cases and smaller coefficients indicating a more equal distribution. In the subfigure A: The Lorenz curves were generated by sorting the GDP per capita in each city/municipal from the lowest to the highest GDP per capita. The x-axis and y-axis represented the cumulative percentage of the population ranked by the GDP per capita and cumulative proportion of notifiable infectious diseases in the surveillance year. In the subfigure B: The Lorenz curves were generated by sorting the urbanization in each city/municipal from the lowest to the highest urbanization. The x-axis and y-axis represented the cumulative percentage of the population ranked by the urbanization and cumulative proportion of notifiable infectious diseases in the surveillance year. (TIF) [file pmed.1004374.s023.tif]

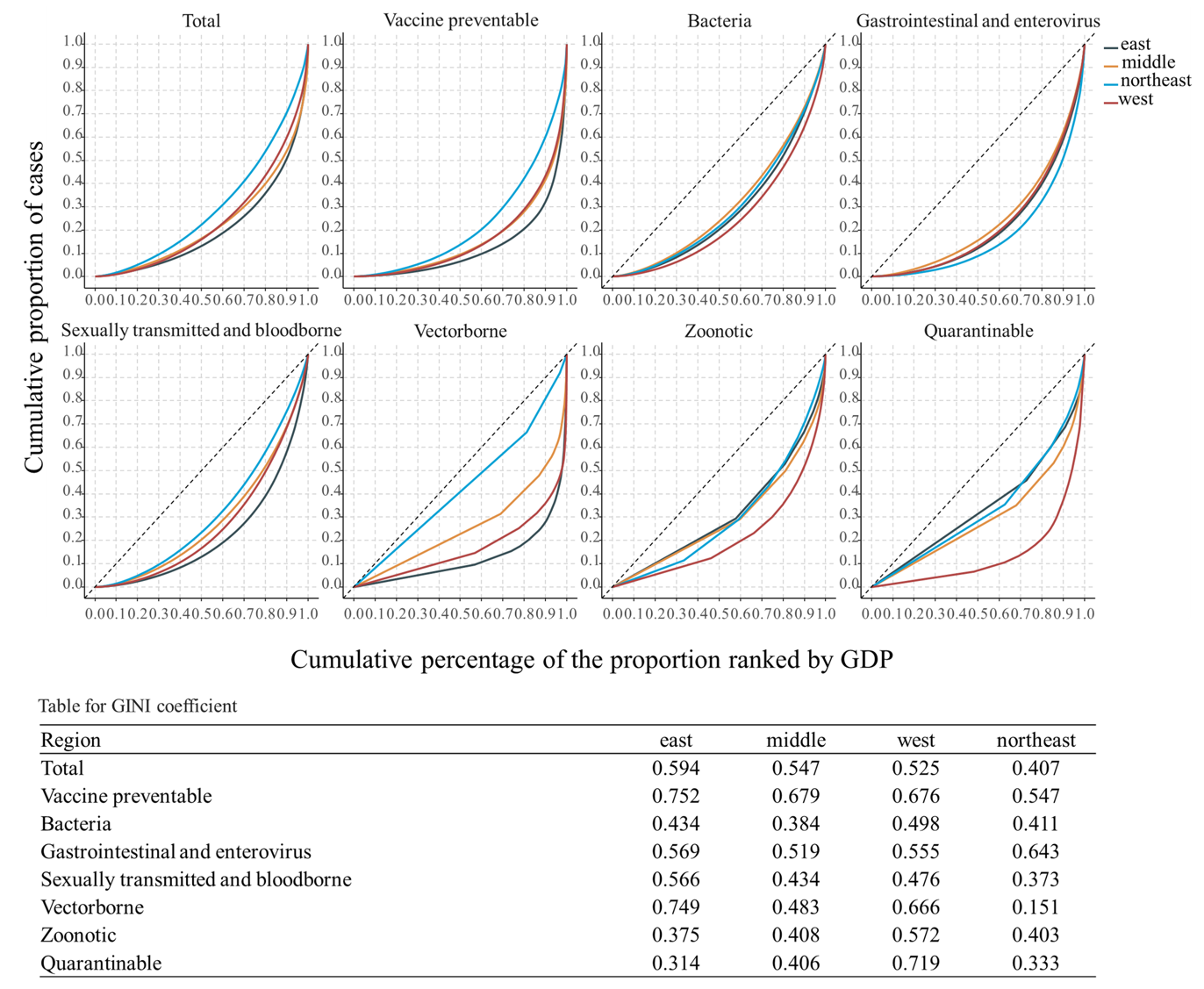

Supplement: S15 Fig — Notes: The (dashed) diagonal line of equality depicted a theoretical scenario where infectious disease cases were equally distributed across the population. The Gini coefficients were calculated based on the Lorenz curves, with larger coefficients indicating more unequal distribution of infectious disease cases and smaller coefficients indicating a more equal distribution. The Lorenz curves were generated by sorting the GDP in each city/municipal from the lowest to the highest GDP. The x-axis and y-axis represented the cumulative percentage of the population ranked by the GDP and cumulative proportion of notifiable infectious diseases in the surveillance year. Table for GINI coefficient shows the GINI coefficient. (TIF) [file pmed.1004374.s024.tif]
